# Mechanism of Multi-Organ Injury in Experimental COVID-19 and Its Inhibition by a Small Molecule Peptide

**DOI:** 10.3389/fphar.2022.864798

**Published:** 2022-05-30

**Authors:** Michael J. Paidas, Natarajan Sampath, Emma A. Schindler, Daniela S. Cosio, Chima Obianuju Ndubizu, Nagarajarao Shamaladevi, Jaclyn Kwal, Suset Rodriguez, Anis Ahmad, Norma Sue Kenyon, Arumugam R. Jayakumar

**Affiliations:** ^1^ Department of Obstetrics, Gynecology and Reproductive Sciences, University of Miami Miller School of Medicine, Miami, FL, United States; ^2^ School of Chemical and Biotechnology, SASTRA Deemed University, Thanjavur, India; ^3^ Molecular Analytics, Miami, FL, United States; ^4^ Department of Radiation Oncology, Sylvester Cancer Center, University of Miami School of Medicine, Miami, FL, United States; ^5^ Microbiology & Immunology and Biomedical Engineering, Diabetes Research Institute, University of Miami, Miami, FL, United States

**Keywords:** animal death, aquaporins, COVID-19, multiorgan failure, oxidative stress, tissue edema, drug treatment

## Abstract

Severe disease from SARS-CoV-2 infection often progresses to multi-organ failure and results in an increased mortality rate amongst these patients. However, underlying mechanisms of SARS- CoV-2-induced multi-organ failure and subsequent death are still largely unknown. Cytokine storm, increased levels of inflammatory mediators, endothelial dysfunction, coagulation abnormalities, and infiltration of inflammatory cells into the organs contribute to the pathogenesis of COVID-19. One potential consequence of immune/inflammatory events is the acute progression of generalized edema, which may lead to death. We, therefore, examined the involvement of water channels in the development of edema in multiple organs and their contribution to organ dysfunction in a Murine Hepatitis Virus-1 (MHV-1) mouse model of COVID-19. Using this model, we recently reported multi-organ pathological abnormalities and animal death similar to that reported in humans with SARS-CoV-2 infection. We now identified an alteration in protein levels of AQPs 1, 4, 5, and 8 and associated oxidative stress, along with various degrees of tissue edema in multiple organs, which correlate well with animal survival post-MHV-1 infection. Furthermore, our newly created drug (a 15 amino acid synthetic peptide, known as SPIKENET) that was designed to prevent the binding of spike glycoproteins with their receptor(s), angiotensin- converting enzyme 2 (ACE2), and carcinoembryonic antigen-related cell adhesion molecule 1 (CEACAM1) (SARS-CoV-2 and MHV-1, respectively), ameliorated animal death and reversed altered levels of AQPs and oxidative stress post-MHV-1 infection. Collectively, our findings suggest the possible involvement of altered aquaporins and the subsequent edema, likely mediated by the virus-induced inflammatory and oxidative stress response, in the pathogenesis of COVID- 19 and the potential of SPIKENET as a therapeutic option.

## Introduction

COVID-19 is caused by infection with SARS-CoV-2, a coronavirus that causes respiratory illness and can ultimately result in organ damage and multi-organ failure (Mokhtari et al., 2020 and references therein). The multi-organ damage is thought to be caused by cytokine storm, septic shock, thrombosis, and oxidative/nitrative stress (Xu et al., 2020). There have been more than 470 million cases in the last 27 months and over 6 million deaths across almost 200 countries. Further, the number of cases and deaths continue to increase despite vaccination, and underlying mechanisms of SARS-CoV-2 induced multi-organ failure and subsequent death remain largely unknown.

The multi-organ dysfunction induced by SARS-CoV-2 includes acute lung, liver and progressive kidney failure, neurological complications, cardiovascular disease, and a multitude of hematologic abnormalities (Gu et al., 2005; Chen et al., 2020; El Zowalaty et al., 2020; Mokhtari et al., 2020; Robba et al., 2020; Shanmugaraj et al., 2020; Zhou et al., 2020; Henderson et al., 2021; Lopes-Pacheco et a., 2021; Matsuishi et al., 2021; Thakur et al., 2021; Tyagi and Singh, 2021; Zhao et al., 2022). The acute lung failure is characterized by the presence of focal, interstitial, diffuse proteinaceous and alveolar edema (Ackermann et al., 2020; Menter et al., 2020). The liver failure is likely due to a direct SARS-CoV-2 cytopathy based on the ultrastructural features of conspicuous mitochondria swelling in the infected hepatocytes (Wang et al., 2020). The progressive kidney injury is due to edema with an associated inflammatory infiltrate of the renal interstitium (Larsen et al., 2020), swelling of cells and edema in the interstitial space of distal tubules and collecting ducts. With regards to neurological complications, autopsies of brain samples from patients who died of COVID-19 revealed the presence of edema (Reichard et al., 2020). Cytotoxic brain edema was also identified in a newborn with COVID-19 (Fragoso et al., 2021). Some reported signs of cardiovascular damage from SARS-CoV-2 infection showed cardiomyocyte hypertrophy, interstitial hyperemia, edema, left ventricular dilatation and inflammation (Puntmann et al., 2020). These findings suggest that the development of edema is multisystemic in SARS-CoV-2 infection.

Cytokine storm has been strongly implicated in the pathogenesis of COVID-19 (Jose and Manuel, 2020; Zaim et al., 2020). One potential consequence of immune and inflammatory events is the acute progression of generalized edema, which may lead to death (Wiggli et al., 2013; Rump and Adamzik, 2018).

Aquaporins (AQPs) are integral membrane proteins that function to aid the diffusion of water and small solutes across the cell membrane, thereby regulating extracellular-intracellular osmolar balance (Verkman and Mitra, 2000; Agre, 2006). Several studies have shown that AQPs can be regulated by microbial/parasitic infections, inflammation-associated responses, and vascular and cell water homeostasis, which implicate their involvement in the disease progression (Azad et al., 2021 and references therein). While thrombosis and inflammation are major factors in SARS-CoV- 2 infection (Mitchell, 2020), studies addressing their interaction with AQPs are absent. Thus far, the only studies that even mention AQPs in relation to respiratory viruses do not investigate the mechanisms: 1) a 59-year-old woman with aquaporin-4-positive neuromyelitis Optica who developed mild respiratory syndrome (Creed et al., 2020), and 2) reduced AQP-1 level was observed in lung endothelial cells in golden Syrian hamsters (Allnoch et al., 2021). However, little to nothing is currently known regarding the role of AQPs in multi-organ dysfunction in SARS- CoV-2 infection. We, therefore, investigated whether AQPs play a role in multi-organ dysfunction in SARS-CoV-2 infection.

We recently established a Murine Hepatitis Virus-1 (MHV-1) mice model of COVID-19 (Paidas et al., 2021). In this model, infection of A/J mice with MHV-1 produces symptoms similar to those seen clinically in patients with SARS-CoV-2 infection and leads to high mortality (Khanolkar et al., 2009; Leibowitz et al., 2010; Agostini et al., 2018; Caldera-Crespo et al., 2021; De Albuquerque., 2006; Paidas et al., 2021; Tian et al., 2021). Two days post-MHV-1 inoculation the mice present with severe pulmonary disease and 7 days post-infection the mortality reaches 60% (Khanolkar et al., 2009; Leibowitz et al., 2010; Agostini et al., 2018; Caldera-Crespo et al., 2021; De Albuquerque., 2006; Paidas et al., 2021; Tian et al., 2021). On day 2, pulmonary edema, as evidenced by fluid accumulation in the alveolar spaces, and patchy interstitial alveolar thickening was observed. On days 2 and 6 post-MHV-1 infection, morphometric analysis showed infiltration of the lung tissue by macrophages and neutrophils. Increased infiltrates of T cells were also noted by day 6 post-infection. At death, the lungs showed severe interstitial pneumonitis with the presence of consolidation, hyaline membranes, fibrin deposition, and dense lymphocyte and macrophage infiltrate. Liver histology was normal up to day 6, but on day 7, just prior to death, there was severe hepatic congestion and lung and heart failure, as described in humans (Agostini et al., 2018; Caldera-Crespo et al., 2021; De Albuquerque et al., 2006; Paidas et al., 2021; Tian et al., 2021). These findings show that A/J mice develop both a SARS-like pulmonary disease and subsequent multi-organ failure when inoculated with MHV-1 intranasally, despite these two coronaviruses using different receptors, angiotensin-converting enzyme 2 (ACE2) and carcinoembryonic antigen-related cell adhesion molecule 1 (CEACAM1) (SARS-CoV-2 and MHV-1, respectively). It should be highlighted that the activation of CEACAM1 was shown to stimulate clinical aspects similar to those of SARS-CoV-2 (Khanolkar et al., 2009; Leibowitz et al., 2010; Agostini et al., 2018; Caldera-Crespo et al., 2021; De Albuquerque et al., 2006; Paidas et al., 2021; Tian et al., 2021).

Coronaviruses have advanced various mechanisms to identify diverse receptors for their cross- species transmission and expansion. These viruses use a variety of cellular receptors and co- receptors that allow them to infect a wide range of avian and mammalian species (Trbojević- Akmačić et al., 2021; Zhang et al., 2021). The spike protein mainly mediates viral entry into host cells (Huang et al., 2020; Trbojević-Akmačić et al., 2021; Zhang et al., 2021), although several other molecules have been suggested (e.g., C-type lectins, DC-SIGN, L-SIGN) (Rahimi, 2020; Thépaut et al., 2021; Jackson et al., 2022). During maturation, the spike protein is cleaved into receptor binding and membrane fusion subunits (S1 and S2 subunits, respectively) (Duan et al., 2020; Huang et al., 2020; Jackson et al., 2022). The S1 subunit contains the amino-terminal (N- terminal) domain (NTD) and C domain, and the C domain binds to the Aminopeptidase-N and ACE2, while the NTD binds to CEACAM1 in MHV (Zhang et al., 2021). However, thus far it is not known whether the NTD plays a functional role in SARS-CoV-2 entry mechanisms.

MHV is more extensively studied than any other coronaviruses. The primary physiological function of its receptor, CEACAM1, is to mediate cell adhesion and signaling. CEACAM1 is chiefly expressed in macrophages, epithelial and endothelial cells. While mammalian CEACAM are conserved, only murine CEACAM1a serves as an efficient MHV receptor (see references Jackson et al., 2022; Peng et al., 2011; Yang et al., 2020 for structural and functional aspects and similarities and differences between ACE2 and CEACAM1). While the molecular determinants for the viral and host specificities of SARS-CoV have been elucidated in the past decade, the interaction between coronaviruses and CEACAM1 remains elusive.

Despite available vaccination against COVID-19, the number of cases and deaths continue to increase. Additionally, the FDA has fully approved just one drug, Remdesivir, to counteract SARS-CoV-2 infection, and only for certain populations (i.e., hospitalized adult and pediatric patients aged ≥12 years and weighing ≥40 kg), while Paxlovid (Nirmatrelvir/Ritonavir tablets- oral use), Dexamethasone, Molnupiravir (MK-4482/EIDD-281), Bamlanivimab, Etesevimab, Casirivimab and Imdevimab were authorized for emergency use by FDA. Based on the molecular structure of SARS-CoV-2 spike glycoprotein-1 (S1) and its interaction with the ACE2 receptor, we designed a peptide (a 15 amino acid synthetic peptide, also known as SPIKENET) that specifically binds to S1, thereby preventing SARS-CoV-2 entry into the host cell. We found that SPIKENET reversed the disease and reduced death in an MHV-1 mice model of COVID-19. SPIKENET also reduced oxidative stress, and altered AQPs and tissue edema in multiple organs, strongly suggesting that infection-induced changes in tissue oxidative stress and the subsequent increase in tissue edema may be crucial factors in the progression of COVID-19.

## Materials and Methods

### SPIKENET, Design, Synthesis and Characterization

Computational protein docking (ClusPro, a protein-protein docking server) was used to design a peptide, SPIKENET that specifically binds to the SARS-CoV-2 spike glycoprotein-1 (S1). We also examined whether SPIKENET has a binding affinity with the human ACE2 receptor. A molecular docking study between the receptor-binding domain (RBD) of SARS-CoV-2 and SPIKENET was performed to understand the nonbonded interactions, and peptide conformation over the human ACE2 binding site of the RBD and to calculate the binding affinity of the peptide. The docking study created 10 different conformations of SPIKENET after the docking simulation. From these conformations, the highest binding affinity model (−156.2 kcal/mol) was chosen for further binding analysis. To confirm the peptide binding affinity of S1 and ACE2 with SPIKENET, we also measured the extent of SPIKENET binding with S1 and ACE2 by spectroscopy. Briefly, the absorbance with relevant wavelength for S1 or ACE2 and SPIKENET was collected independently as well as when S1 or ACE2 and SPIKENET were added together.

Since the MHV-1 host receptor is CEACAM1 (CCM), we first examined whether SPIKENET has a binding affinity with the CEACAM-1 RBD of the MHV-1 N-terminal domain (NTD) (MHV-1 S1) as well as with the CCM. Accordingly, we performed molecular docking of SPIKENET with SARS-CoV-2 S1, as well as molecular dynamic studies to confirm the binding of SPIKENET with MHV-1 NTD, and with CCM.

The initial structures of the MHV1-NTD and CCM human receptor were obtained from the Protein Data Bank database from the structure of 6vsj, and the NTD was truncated at residues 16–283 from MHV1-S1 for dynamic study. The ligand peptide of SPIKENET has 15 residues with the sequence of MVRIKPASANKPSDD and its 2D structure was built using the Avogadro (v. 1.2.0) molecular modeling program, and geometrical optimization was done using the Steepest Descent algorithm with the universal force field to get an energy-optimized 3D structure. To investigate the binding efficacy of SPIKENET with the NTD of MHV1-S1 and receptor CCM, the truncated NTD and CCM were docked with SPIKENET using ClusPro server at the NTD and CCM binding site. SPIKENET binding with NTD and CCM was subjected to Molecular dynamics (MD) simulation to understand the binding affinity of SPIKENET (SPK) with NTD and CCM. MD simulation was performed with the default setting of the GROMACS 5.4.1 package and simulation was done by applying GROMOS96 54a7 force field for all protein atoms. After the minimization of complex structure, position restrained MD simulation was done upon slow heating to 300 K in NVT [conserved amount of substance (N), volume (V) and temperature (T) in the canonical ensemble] and NPT [conserved amount of substance (N), pressure (P) and temperature (T) in the isothermal–isobaric ensemble] with a constant particles number, constant temperature, constant volume, and constant pressure, respectively, at 1 atm pressure throughout 500 ps. The final production MD calculation was then carried out for a total of 50ns MD simulation for our target complexes of NTD + SPK and CCM + SPK with a time step of 1 fs at the constant pressure (1 atm) and temperature (300 K). The MD trajectories were analyzed from time to time using the visual molecular dynamic (VMD) program.

### MHV-1 Inoculation and the Effect of SPIKENET

To test the effect of our peptide on SARS-CoV-2 infection, we used an established mice model of COVID-19 (Agostini et al., 2018; Caldera-Crespo et al., 2021; De Albuquerque., 2006; Paidas et al., 2021; Tian et al., 2021). Female A/J mice (8 weeks old, weighing 22 g) were purchased from Jackson Laboratories (Bar Harbor, ME) and were housed in pairs in micro-isolated cages in the animal colony at the Biomedical Research Building animal isolation facility at the University of Miami Miller School of Medicine. Mice were fed standard lab chow and provided with water ad libitum. The study followed the guidelines of the University of Miami Institutional Animal Care and Use Committee (IACUC protocol number 20-131 LF) approved on 8 October 2020.

MHV-1 was purchased from American Type Culture Collection (ATCC, cat# VR-261, Manassas, VA). Mice were inoculated with 5,000 PFU intranasally (Agostini et al., 2018; Caldera-Crespo et al., 2021; De Albuquerque et al., 2006; Mokhtari e al., 2020; Paidas et al., 2021; Tian et al., 2021) which was produced by mixing 5 × 10^3^ PFU MHV-1 with 50 μl of ice-cold Dulbecco’s modified eagle medium (DMEM) and instilled into the nares as quickly as possible. The mice were observed to ensure inhalation of the virus. The mice were divided into five groups: 1) healthy controls, 2) infusion of healthy controls with DMEM, 3) infusion of MHV-1 alone, 4) infusion of SPIKENET alone, and 5) infusion of MHV-1 + SPIKENET (5 mg/kg. b.wt.).

### Clinical Observation

A/J mice inoculated with MHV-1 with and without SPIKENET were monitored for clinical signs as described previously (Agostini et al., 2018; Caldera-Crespo et al., 2021; De Albuquerque et al., 2006; Paidas et al., 2021; Tian et al., 2021). Symptoms were scored by stages: 0) without symptoms, I) drowsiness and lack of motion, II) slightly ruffled fur and altered hind limb posture, III) ruffled fur and difficulty breathing, IV) ruffled fur, inactive, moderately labored breathing, V) ruffled fur, severe difficulty breathing and lethargy, and VI) moribund and death (Tian et al., 2021).

Mice that reached a disease stage of V and VI were weighed and euthanized, and their organs were removed and fixed in 10% formalin, processed routinely for paraffin sections and stained with Hematoxylin and Eosin. To measure the extent of liver failure, blood was collected via cardiac puncture once mice reached stages V and VI and serum was used to measure aspartate aminotransferase (AST), alanine aminotransferase (ALT), alkaline phosphatase (ALP), and bilirubin, with and without SPIKENET, as previously described (Paidas et al., 2021; Tian et al., 2021). Post-MHV-1, body weight was measured daily in mice both with and without SPIKENET treatment.

### Examination of Viral Proteins and AQPs in Tissues by Immunohistochemistry (Immunofluorescence)

Paraffin-embedded tissue sections from normal (uninfected) and MHV-1-inoculated mice (10 microns) with and without SPIKENET were incubated with antibodies specific to viral proteins and AQPs SARS-CoV-2 (COVID-19) Spike S1 rabbit monoclonal antibody (Cat# GTX635671; GeneTex California, United States); and SARS-CoV-2 Nucleocapsid mouse monoclonal antibody (Cat# MA5-29981: Invitrogen Massachusetts, United States) were used at 1:200 dilution. AQP1 Polyclonal Antibody (Cat# A53228-020: EpiGentek NY, United States); AQP4 Polyclonal Antibody (Cat# A50672- 020: EpiGentek NY, United States); AQP5 Polyclonal Antibody (Cat# BS-1554R-TR: Bioss Antibodies, Massachusetts, United States); and AQP8 Polyclonal Antibody (Cat# PA1511; Boster Biological Co., Ltd. California, United States) were used at 1:300 dilution. Horseradish peroxidase-conjugated anti-rabbit and anti-mouse secondary antibodies (Vector Laboratories CA, United States) were used at 1:500 dilution. Immunofluorescent images were acquired with a Zeiss LSM510/UV Axiovert 200 M confocal microscope (Carl Zeiss Microscopy, LLC, Thornwood, NY, United States) with a plan apochromat ×40 objective lens, and ×2 zoom resulting in images of 125 × 125 μm in the area and 1.0 μm optical slice thickness (1.0 Airy units for Alexa Fluor 546 or 568 emission channel). The images were randomly collected in a “blinded” manner. At least 12 fluorescent images were captured per mouse. Images were quantified using the Volocity 6.0 High-Performance Cellular Imaging Software (PerkinElmer, Waltham, MA, United States) as described previously (Jayakumar AR. et al., 2014; 2017), and normalized to the number of DAPI-positive cells and to the area and intensity of DAPI.

### Immunoblot

Tissues from uninfected, infected, and drug-treated animals, were subjected to gel electrophoresis and immunoblotting as described previously (Jayakumar AR. et al., 2014; Jayakumar et al., 2014 A. R.). Primary antibodies to AQP1 Polyclonal Antibody (Cat# A53228-020: EpiGentek NY, United States); AQP4 Polyclonal Antibody (Cat# A50672-020: EpiGentek NY, United States); AQP5 Polyclonal Antibody (Cat# BS-1554R-TR: Bioss Antibodies, Massachusetts, United States); AQP8 Polyclonal Antibody (Cat# PA1511; Boster Biological Co., Ltd. California, United States) and β-actin mouse monoclonal (C4, Cat# sc-47778: Santa Cruz Biotechnology, Inc. California, United States), were used at 1:2,000 dilution. Horseradish peroxidase-conjugated anti-rabbit and anti-mouse secondary antibodies (1:1,000) (Vector Laboratories, CA, United States) were used at 1:5,000 dilution. Optical density of the bands was quantified with the Sigma Scan Pro program (Jandell Scientific, CA, United States) as a proportion of the signal of a house-keeping protein band (β-actin) (Jayakumar AR. et al., 2014; Jayakumar et al., 2014 A. R.).

### Tissue Water Measurement

Tissue water content was measured by the wet/dry weight method (Jayakumar AR. et al., 2014; Jayakumar et al., 2014 A. R.) where approximately 10 mg of tissue (8 pieces from each mouse) was dissected, and wet weights of tissue were determined. The tissue was then dried in an oven at 100°C and dry weights were determined. The difference in wet/dry weights was expressed as percent water content.

### Primary Culture of Astrocytes

Primary cultures of cortical astrocytes were prepared as described previously (Ducis et al., 1990). The cerebral cortices of 1-2-day-old rat pups were minced and homogenized, filtered, and the pellet was re-suspended and seeded onto 35 mm culture dishes in DMEM containing penicillin, and streptomycin and 15% fetal bovine serum. The culture plates were then incubated at 37°C with 5% CO_2_ and 95% air, ensuring changing of the culture media twice weekly. Fetal bovine serum was replaced with 10% horse serum 10 days after seeding. After14 days, cultures were treated with 0.5 mm dibutyryl cAMP (Sigma, St. Louis, MO, United States) to enhance cellular differentiation (Juurlink and Hertz, 1985). Cultures were determined to have at least 95% astrocytes as determined by glial fibrillary acidic protein (GFAP) immunohistochemistry. All cultures used were 24–28 days old.

### Primary Culture of Microglia

Primary cultures of rat microglia were grown on a monolayer of astrocyte cultures prepared from brains of 1-day old pups following the method of Flanary and Streit (Flanary and Streit, 2006). The meninges of the cerebral cortices were removed and the cerebral cortices were minced in Hank’s balanced salt solution (0.137 M NaCl, 0.2 M NaH2PO4, 0.2 M KH2PO4, 5.4 mm KCl, 5 mm glucose, 58.4 mm sucrose, 0.25 μg/ml Fungizone, and 1 × 106 U penicillin/streptomycin) with 0.25% trypsin and incubated for 30 min at 37°C. 5 ml of DMEM containing 10% fetal bovine serum (FBS) and 1% penicillin/streptomycin was used to stop the trypsin reaction. The suspension was triturated multiple times and passed through sterile filters (130, 40 μm). The suspension was then ultimately seeded into T75 flasks and allowed to grow for 4 days, at which point the medium was changed and incubation was continued for an additional 6 days. Flasks were shaken on an orbital shaker (100 rpm) for 1 h at 37°C and the media containing microglia was then collected, centrifuged and replated at a density of 1 × 104 in 35 mm^2^ plates. ED1 immunoreactivity was used to ensure that the cultures were composed of at least 98% microglia.

### Statistical Analysis

*In vivo studies*: Five to sixteen mice from control and experimental groups were presented. The data were subjected to analysis of variance followed by Tukey’s multiple comparison test. A *p* < 0.05 value was considered significant. *In vitro studies*: Each group consisted of 5 culture dishes per experiment for free radical, LDH release and protein carbonyl measurements, and four culture dishes each per experiment for cell volume and cytokine measurements, respectively. Each set of experiments was performed 5–11 times from multiple seedings. The extent of cell swelling, and other analysis were normalized to protein values and subjected to analysis of variance (ANOVA) followed by Tukey’s post-hoc comparisons.

## Result

### SPIKENET Synthesis and Binding Affinity to the Spike Glycoprotein

**FIGURE 1:**
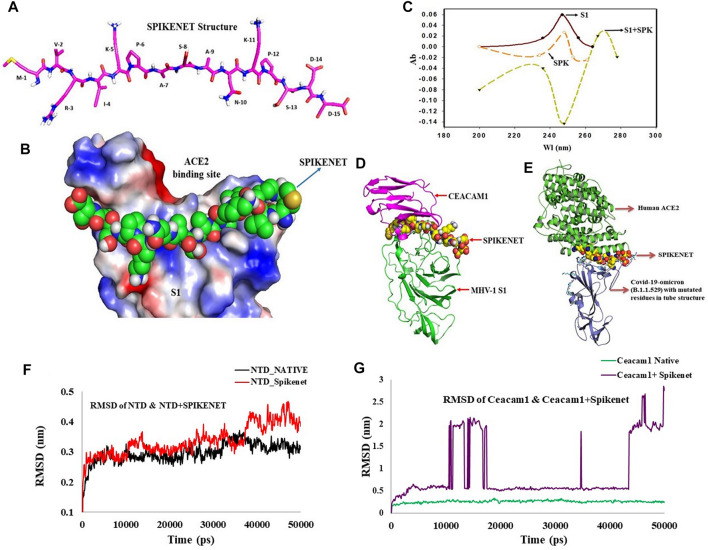
SPIKENET Structure and Binding Affinity to the ACE2 Binding Domain of the SARS-CoV-2 Spike Glycoprotein. **(A)** Structure of SPIKENET, a 15 amino acid synthetic peptide targeted to the ACE2 binding domain of the SARS-CoV-2 spike glycoprotein. **(B)** Computational protein docking approach shows highly specific SPIKENET binding affinity to the ACE2 binding domain of the SARS-CoV-2 spike glycoprotein (S1). **(C)** Spectroscopic analysis shows highly specific SPIKENET (SPK) binding affinity to the ACE2 binding domain of the SARS-CoV-2 spike glycoprotein (S1). **(D)** High affinity binding of SPIKENET to the CEACAM1 binding domain of the MHV-1 spike glycoprotein: Computer modeling studies. **(E)** highly specific SPIKENET binding affinity to S1-RBD of the recently identified Omicron SARS-CoV-2 variant. Ab, absorbance; Wl, wavelength. High affinity binding of SPIKENET to the CEACAM1 binding domain of the MHV-1 spike glycoprotein. High affinity binding of SPIKENET to the CEACAM1 binding domain of the MHV-1 spike glycoprotein: Computer modeling studies. Ab, absorbance; Wl, wavelength. High affinity binding of SPIKENET to the CEACAM1 binding domain of the MHV-1 spike glycoprotein: Confirmatory computer molecular dynamic studies. The RMSD of both complexes with their respective native proteins are shown in **(F,G)**. Comparing the RMSD of both NTD and NTD + SPK **(F)** after 50 ns dynamic simulation, the structures showed complete equilibration in the system and the SPK peptide was well stabilized with high affinity at the CCM binding location of NTD. However, the RMSD analysis of CCM and CCM+ SPK structures after the 50 ns dynamic simulation exhibited more flexibility at the NTD binding site of CCM than native CCM **(G)**, suggesting the SPK peptide detachment and displacement over the CCM.

Since no drugs currently exist to effectively eradicate SARS-CoV-2 infection, identification of a therapy to combat SARS-CoV-2 is extremely critical. We found highly specific SPIKENET binding affinity to the human ACE2 binding domain of spike glycoprotein-1 (S1) as determined by computational protein docking and by spectroscopy (Figures 1A–C).

Since we use the MHV-1 mouse model of COVID-19 (Biosafety Level-2 facility), we examined whether SPIKENET (SPK) has similar binding affinity with MHV-1 S1. We now show SPK binding affinity to the CCM binding domain of the MHV-1 NTD by modelling (Figure 1D), as well as by molecular dynamic studies (Figures 1F,G). Briefly, all the trajectories of both complexes, N terminal domain-SPIKENET (NTD-SPK) and CEACAM1-SPIKENET (CCM-SPK), were analyzed to understand the binding affinity of SPK with NTD/ CCM and their conformational changes during the simulation. Hence, the root mean square deviation (RMSD) for protein backbone atoms using least-squares fitting, and the root mean square fluctuation for every residue were calculated for both complexes and the native proteins using their final coordinates obtained from MD simulation. The RMSD of both complexes, NTD-SPK and CCM-SPK, with their respective native proteins are shown in Figures 1F,G. Comparing the RMSD of both NTD/NTD + SPK after 50 ns dynamic simulation, the structures showed complete equilibration in the system and the SPK peptide is well stabilized with high affinity at the CCM binding location of the NTD (Figure 1F). However, the RMSD analysis of CCM and CCM + SPK structures after the 50 ns dynamic simulation exhibit more flexibility at the NTD binding site of CCM than native CCM (Figure 1G), suggesting SPK detachment/displacement over the CCM. The initial detachment of SPK showed in the range of 10–18 ns, and after 45 ns SPK had completely dissociated from the CCM. The stabilized RMSD of NTD with SPK indicates the reliability of peptide binding with the NTD. Noteworthy, in addition to a highly specific SPIKENET binding affinity to the ACE2 binding domain of spike glycoprotein-1 (S1), we also found highly specific SPIKENET binding affinity to S1-RBD of the recently identified Omicron SARS-CoV-2 variant (Figure 1E), a s well as with other SARS-CoV-2 variants (alpha, beta, gamma, delta variants, data not shown).

**FIGURE 2:**
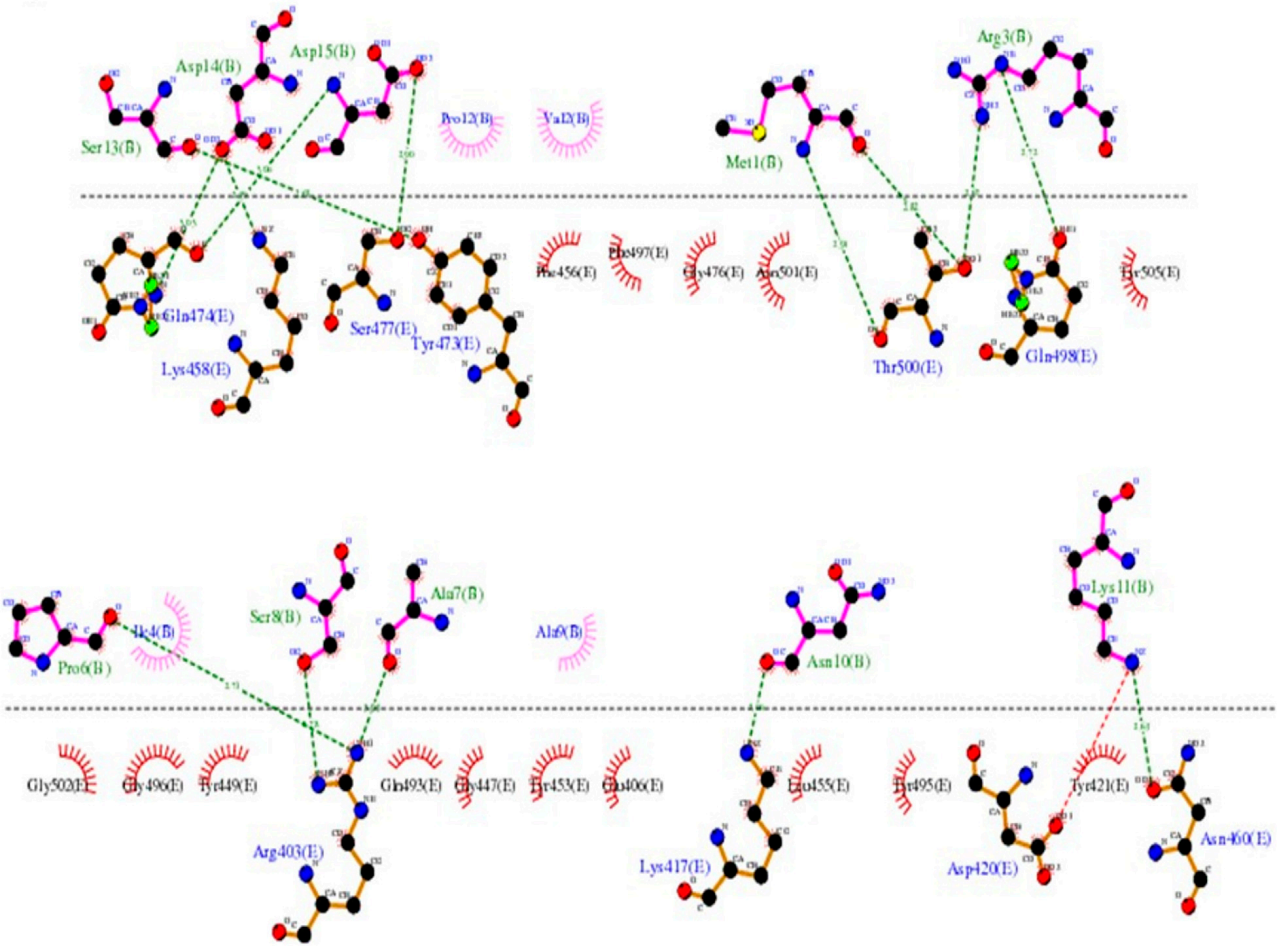
The LIGPLOT diagram of protein-peptide interaction between the RBD and SPIKENET. The pink color SPIKENET peptide residues are shown on the top and yellow color amino acid residues of the RBD from SARS-CoV- two are shown at the bottom. The SPIKENET peptide consists of 15 amino acid residues, 14 of which are shown in non-bonded interactions with the RBD of SARS-CoV-2, which proves that SPIKENET has a significant binding affinity with the spike glycoprotein-1. These findings strongly suggest that SPIKENET is a potent competitive inhibitor of S1.

The LIGPLOT diagram of protein-peptide interaction between the RBD and SPIKENET is shown in Figure 2. The pink color SPIKENET peptide residues are shown on the top and yellow color amino acid residues of the RBD from SARS-CoV-2 are shown at the bottom. The SPIKENET (>98.7% pure by HPLC analysis and no other traces identified) peptide consists of 15 amino acid residues, 14 of which are shown in non-bonded interactions with the RBD of SARS-CoV-2, which proves that SPIKENET has a significant binding affinity with the S1. These findings strongly suggest that SPIKENET is a potent competitive inhibitor of S1 that can effectively prevent SARS-CoV-2 endocytosis.

As reported in several structures, the residues which are involved in binding with the human ACE2 receptor, leading to viral infection in humans, are found to be similar to those of SPIKENET peptide binding. Binding analysis of the complex structure of RBD-SPIKENET shows that the residues Gln-474, Lys-458, Ser-477, Tyr-473, Thr-500, Gln-498, Arg-403, Lys-417, Asp-420, and Asn-460 from the RBD have strong electrostatic intermolecular interactions (within the range of 2.59–3.06 A distances) with the residues Ser-13, Asp-14, Asp-15, Met-1, Arg-3, Pro-6, Ser-8, Ala-7, Asn-10, and Lys-11 from SPIKENET. Apart from these non-bonded interactions in this complex, the residues Phe-456, Phe-497, Gly-476, Asn-501, Tyr-505, Gly-502, Gly-496, Tyr-449 Gln-493, Gly-447, Tyr-453, Glu-406, Leu-455, Tyr-495, and Tyr-421 show hydrophobic interactions with residues Pro-12, Val-2, Ile-4, and Ala-9 from SPIKENET, which contribute significantly to peptide stability at the ACE2 binding site of the RBD.

Multiple sequence alignment (MSA) of human receptor binding domains (RBDs) from SARS- CoV-2 and N-terminal domain (NTD) from MHV-1 confirms that both these domains share less than 12% sequence similarity, which suggests that both these structures are dissimilar. Moreover, the structural alignment of the RBDs and NTDs of viral spike proteins showed a root mean square deviation (RMSD) of 17.4 Å, which also strongly supports the structural dissimilarity, but they hold the β-sheets in their core structure. Interesting to note, the investigation of both human receptor binding sites by the creation of an electrostatic surface diagram revealed that the structures are dissimilar, but receptor binding sites of both the structures have almost similar, highly hydrophobic binding environments. Hence the ability of both these domains to strongly bind with SPIKENET.

**FIGURE 3:**
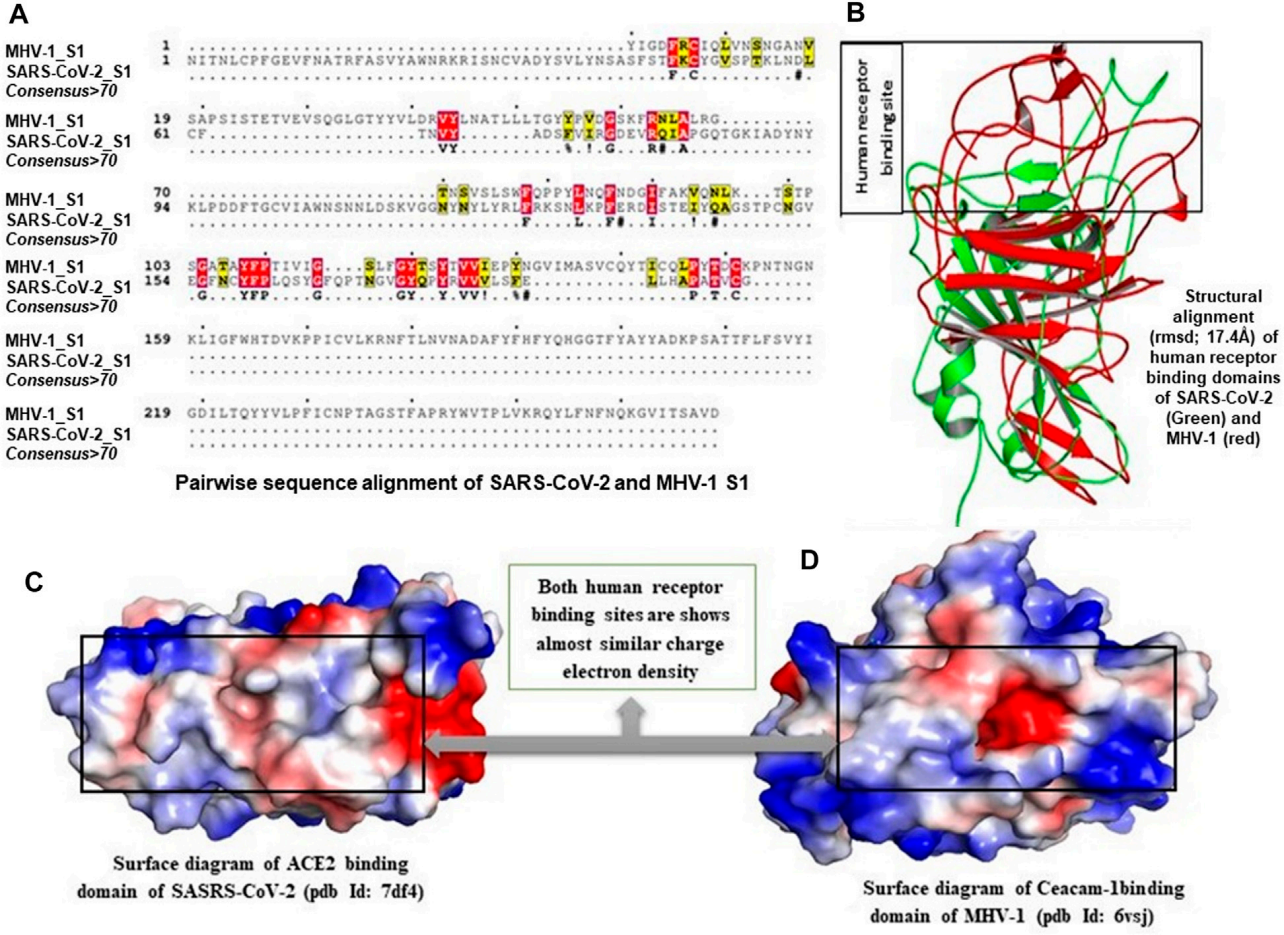
Multiple sequence alignment (MSA) of human receptor binding domains (RBDs) from COVID-19 and N-terminal domain (NTD) from MHV-1 are shown in **(B)**, which confirms that both these domains share less than 12% sequence similarity, which suggests that both these structures are dissimilar. Moreover, structural alignment of the RBDs and NTDs of viral spike proteins showed a root mean square deviation (RMSD) of 17.4 Å **(A)**, which also strongly supports the structural dissimilarity, but they hold the β-sheets in their core structure. Interesting to note, the investigation of both human receptor binding sites by the creation of an electrostatic surface diagram revealed that the structures are dissimilar, but receptor binding sites of both the structures have almost similar, highly hydrophobic binding environments **(C,D)**. Hence the ability of both these domains to strongly bind with SPIKENET.

While it is unclear how SPIKENET has a high binding affinity with S1 proteins of both SARS- CoV-2 and MHV-1, we examined multiple sequence alignments (MSA) of human RBDs from SARS-CoV-2 and NTD from MHV-1 receptor binding sites by creating an electrostatic surface diagram. While we found that the structures are different, the RBDs of both the structures have similar, highly hydrophobic binding environments (Figure 3), resulting in strong binding affinity with SPIKENET. The theoretical mass of SPIKENET is 1628.9 Da and the observed mass is 1628.4 Da. The final purity of the peptide is 98.7%. The peptide was synthesized by Sigma/Aldrich (Saint Louis, MO, United States). It is supplied in 10 mg vials of a white lyophilized powder. It does not contain any preservative, and is chemically and physically stable for 72 h after reconstitution when stored at −20 to −40°C. The lyophilized powder can be stored at −20 to −80°C. For short-term (3–6 months) the powder can be stored at −20°C and for long-term (6–48 months) the powder can be stored at −80°C. The specific effective shelf life can be obtained from the product information sheet. The peptide was not administered after the expiration date indicated on the package and vial. To ensure product sterility, the peptide was reconstituted and administered using aseptic techniques.

### Effect of SPIKENET on MHV-1 Inoculated Mice

**FIGURE 4:**
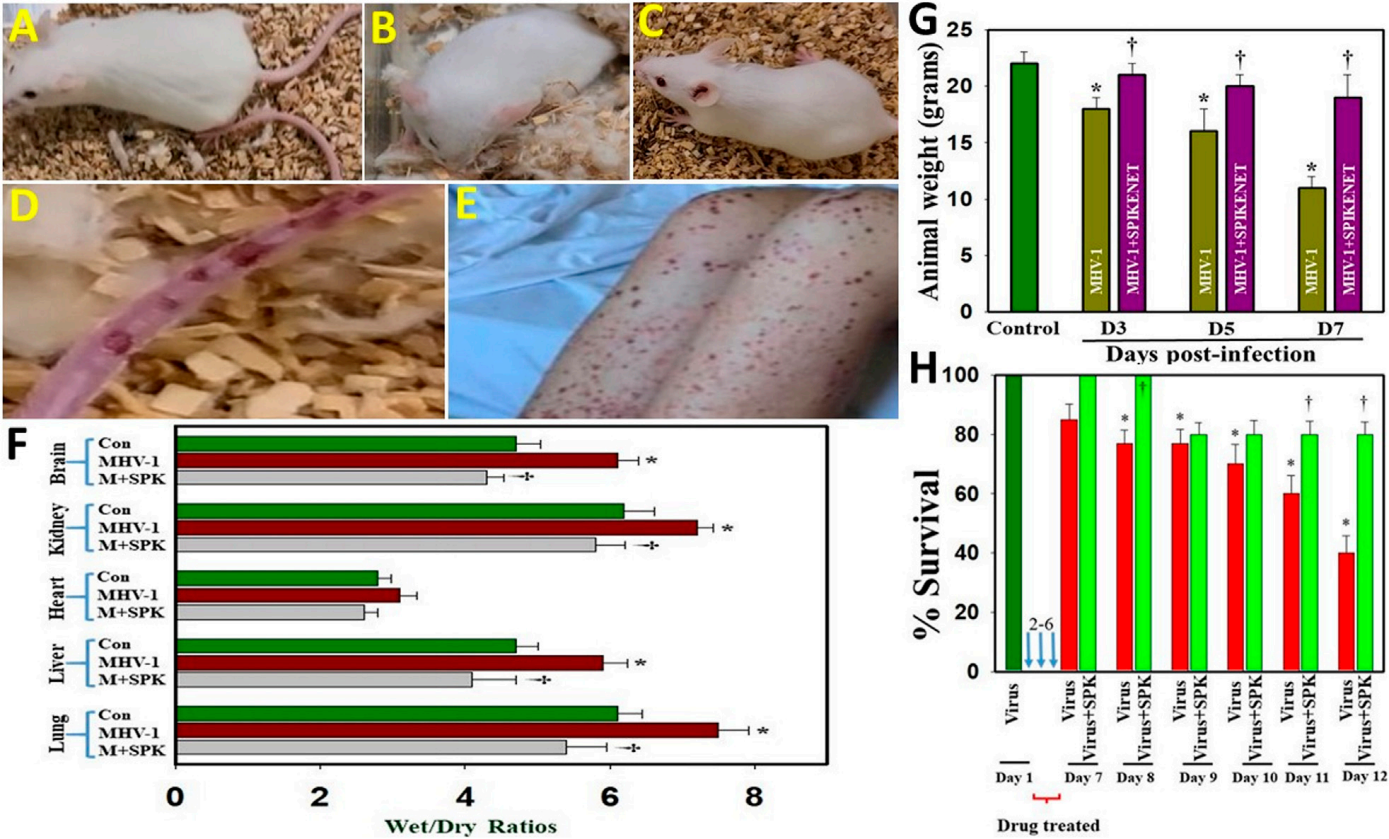
Severe sickness in MHV-1 inoculated mice. **(A)** Normal mouse. **(B)** MHV-1 infected mice showing sickness. **(C)** MHV-1 inoculated mice treated with 5 mg/kg SPIKENET (SPK) ameliorated the clinical symptoms and disease progression. **(D)** MHV-1 infected mice showing venous thrombosis similar to symptoms in patients with COVID-19 **(E)**. Elevated edema was observed in MHV-1 infected brain, lung, liver, kidney, and heart, as compared to control **(F)**. Treatment of MHV-1-infected mice with SPK (5 mg/kg; 3 injections from 2 to 6 days) showed edema level similar to control on day 7 **(F)**. SPK also reversed MHV-1-induced reduction in animal weight **(G)**, as well as improved survival **(H)**. ANOVA, *n* = 5 for control; *n* = 16 for virus alone and *n* = 7 for MHV1 + SPK group (similarly for Figures 5, 6 (see below). *p < 0.05 versus control; †*p* < 0.05 verses MHV-1 infected mice. Con, control. Error bars represent mean ± SEM.

We earlier reported that MHV-1 inoculated mice displayed severe venous thrombosis, body weight loss and death (Agostini et al., 2018; Caldera-Crespo et al., 2021; De Albuquerque et al., 2006; Paidas et al., 2021; Tian et al., 2021), characteristic features of COVID-19. We now found that treatment of MHV-1 inoculated mice with SPIKENET (3 doses of 5 mg/kg, given every alternate day from day 2, i.e., 2, 4 and 6 days post-MHV-1) diminished the formation of venous thrombosis (Figure 4C), body weight loss (Figure 4G), edema and death rate (Figures 4F–H). We also found increased liver enzymes (AST and ALT) in MHV-1-infected mice and that SPIKENET prevented these changes (AST, 3459.2 ± 684.1 units/l in MHV-1 infected mice, as compared to 96.8 ± 14.2 in control and 608.7 ± 356.8 in SPK treated group; ALT, 3068.5 ± 861.3 units/l in MHV-1 infected mice, as compared to 31.5 ± 11.6 in control and 1029.1 ± 436.7 in SPK treated group; ALP, 986.3± 158.4 units/l in MHV-1 infected mice, as compared to 589.1 ± 108.7 in control and 643.8 ± 94.1 in SPK treated group; billirubin, 0.86 ± 0.2 units/l in MHV-1 infected mice, as compared to 0.075. ± 0.02 in control and 0.23 ± 0.06 in SPK treated group).

### SPIKENET Diminished Edema in the MHV-1 Mouse Model of COVID-19

Studies in humans associated with SARS-CoV-2 infection suggest the probable role of generalized edema in the disease progression of COVID-19 (Lopes-Pacheco et al., 2021 and references therein). Since these studies are based on histopathological examinations, we utilized our MHV-1 mouse model to investigate whether generalized edema also occurrs and the means by which MHV-1 induces edema. We identified increased edema with varying degrees in lung, liver, brain, kidney and heart (Figure 4F). Treatment of MHV-1-inoculated mice with SPIKENET showed an edema level similar to control on day 7. Further, treatment of MHV-1-inoculated mice with a small molecular peptide (VRIKPGTANKPSED) had no effect in animal survival consistent with lack of binding affinity with S1 or ACE2/CEACAM1 (Figure not shown). These findings suggest that the development of edema in various organs may be a critical event in SARS-CoV-2 infection, and that our newly created peptide, SPK, which was effective in preventing S1 binding with ACE2 or CCM, offers a potential therapeutic strategy for SARS-CoV-2 infection.

### Histopathological Changes in a Mouse Model of COVID-19

**FIGURE 5:**
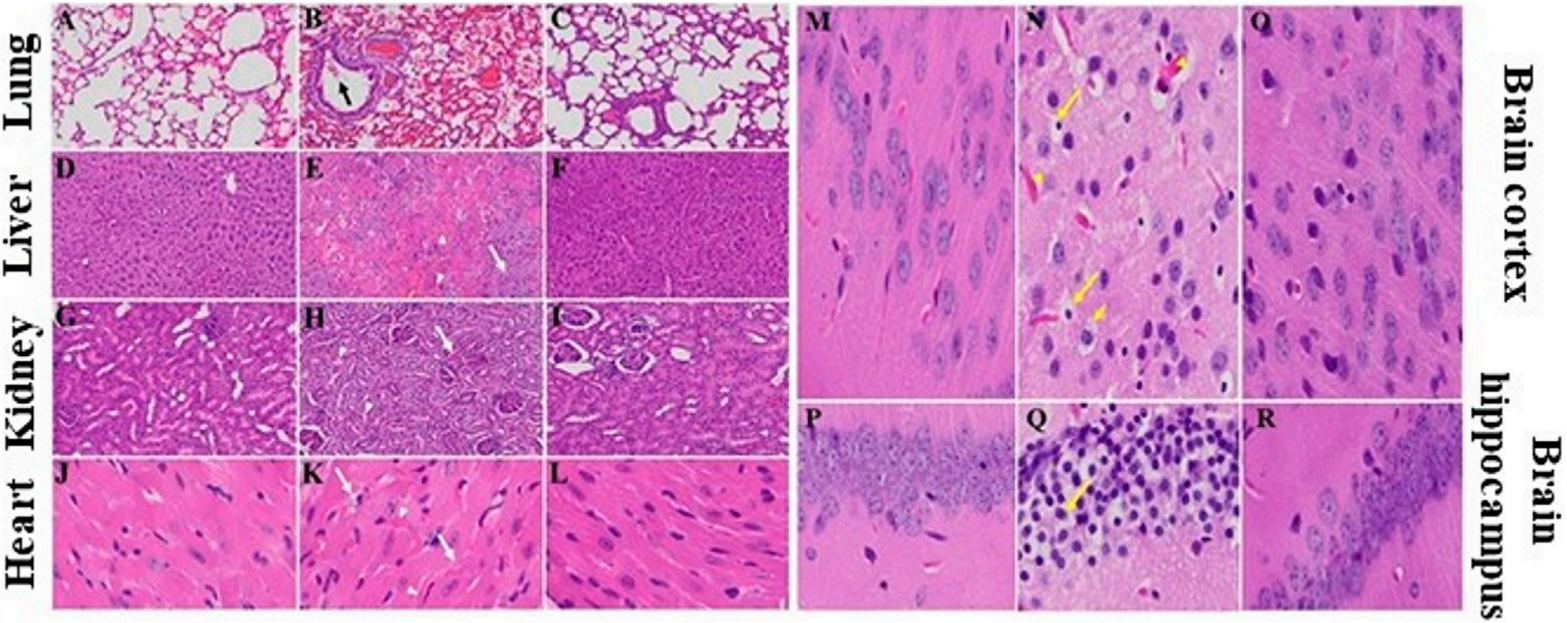
SPIKENET (SPK) diminishes MHV-1-induced pathological changes in lung, liver, kidney, heart and brain. Representative histological images of hematoxylin and eosin (H&E) stained lung, liver, kidney, heart and brain tissue sections of a normal mouse **(A,D,G,J,M,P)** and an infected mouse at day 7 **(B,E,H,K,N,Q)**. “MHV-1-infected mice lungs showed arterial endothelial swelling, inflammation/granular degeneration of cells and migration of leukocytes into lung (arrow). Peribronchiolar interstitial infiltration, bronchiole epithelial cell necrosis and necrotic cell debris within alveolar lumens, alveolar exudation, infiltration, hyaline membrane formation and alveolar hemorrhage with red blood cells within the alveolar space and interstitial edema were also observed in MHV-1-infected mice **(B)**. MHV-1-infected mice at day 7 showed hepatocyte degeneration, severe periportal hepatocellular necrosis with pyknotic nuclei, severe hepatic congestion (arrowheads), ballooned hepatocytes (arrow), vacuolation and the presence of piecemeal necrosis, as well as hemorrhagic changes and hemorrhagic changes when compared to uninfected mice **(E)**. Kidney from an MHV-1 infected mouse showed proximal and distal tubular necrosis (arrowheads), hemorrhage in the interstitial tissue (arrow), and vacuolation of renal tubules **(H)**. MHV-1 infected mouse heart showed severe interstitial edema (arrows), vascular congestion and dilation (arrowheads), and red blood cell extravasation into the interstitium **(K)**. Congested blood vessels (arrowhead), pericellular halos (short arrow), pyknotic nuclei amid associated vacuolation of the neuropil (long arrows), perivascular cavitation suggestive of edema, vacuolation of neuropils, darkly stained nuclei, and acute eosinophilic necrosis were observed in MHV- 1-treated mice **(N,Q)**, as compared to untreated mice” (Paidas et al., 2021). MHV-1 inoculated mice treated with 5 mg/kg SPIKENET (SPK) ameliorated all of these changes **(C,F,I,L,O,R)** (H&E original magnification is ×400 for all images).

We recently showed that MHV-1 inoculated mice displayed weight loss and death (Agostini et al., 2018; Caldera-Crespo et al., 2021; De Albuquerque et al., 2006; Paidas et al., 2021; Tian et al., 2021), just like that seen in humans with SARS-CoV-2 infection. Additionally, multi-organ histopathological damage similar to that observed in humans with SARS-CoV-2 was observed in the MHV-1 inoculated mice. Lung findings include “severe lung inflammation, peribronchiolar interstitial infiltration, bronchiolar epithelial cell necrosis, intra-alveolar necrotic debris, alveolar exudation, mononuclear cell infiltration, hyaline membrane formation, the presence of hemosiderin-laden macrophages, as well as interstitial edema” (Paidas et al., 2021). The livers of infected mice showed “severe liver vascular congestion, luminal thrombosis of portal and sinusoidal vessels, hepatocyte degeneration, cell necrosis and hemorrhagic changes” (Paidas et al., 2021). In the kidney there was “proximal and distal tubular necrosis, hemorrhage in interstitial tissue, and vacuolation of renal tubules” (Paidas et al., 2021). Examination of the heart demonstrated “severe interstitial edema, vascular congestion and dilation, and red blood cell extravasation into the interstitium” (Paidas et al., 2021). The MHV-1 infected mice brains were riddled with “congested blood vessels, perivascular cavitation, cortical pericellular halos, vacuolation of neuropils, darkly stained nuclei, pyknotic nuclei and associated vacuolation of the neuropil in the cortex, and acute eosinophilic necrosis and necrotic neurons with fragmented nuclei and vacuolation in the hippocampus” (Paidas et al., 2021). Noteworthy, treatment of MHV-1 infected mice with 5 mg/kg SPIKENET reversed these changes (Figure 5).

### Altered AQPs in Multiple Organs Post-MHV-1-Inoculation in Mice

**FIGURE 6:**
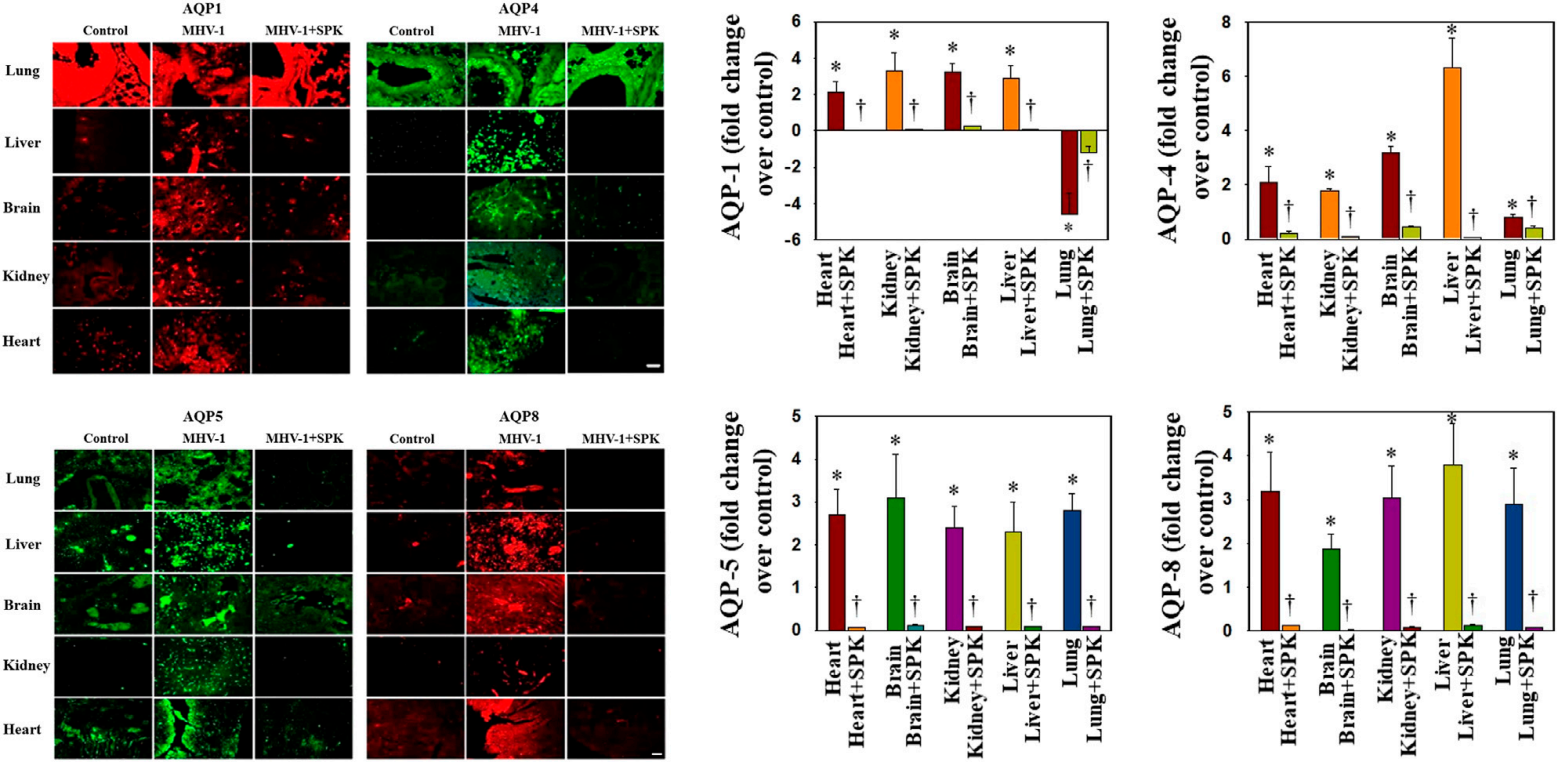
Altered AQP levels were identified in various organs post-MHV-1 inoculation. While increased AQPs 1 and 4 have been identified in various organs, AQP1 levels were decreased in lungs post-MHV-1. Further, treatment of MHV-1 inoculated mice with SPK (5 mg/kg) reversed these changes. A widespread increase in AQPs 5 and 8 was observed in mice post-MHV-1 inoculation, and treatment of MHV-1 inoculated mice with SPK (5 mg/kg) reversed these changes. Scale bar = 25 μm. Quantitation of AQPs 1, 4, 5, and 8 immunofluorescence images. ANOVA, *n* = 5 (Control), 16 (MHV-1) and 7 (MHV-1 + SPK). *p < 0.05 versus control; ^†^ *p* < 0.05 verses MHV-1 infected mice. Error bars represent mean ± SEM.

**FIGURE 7:**
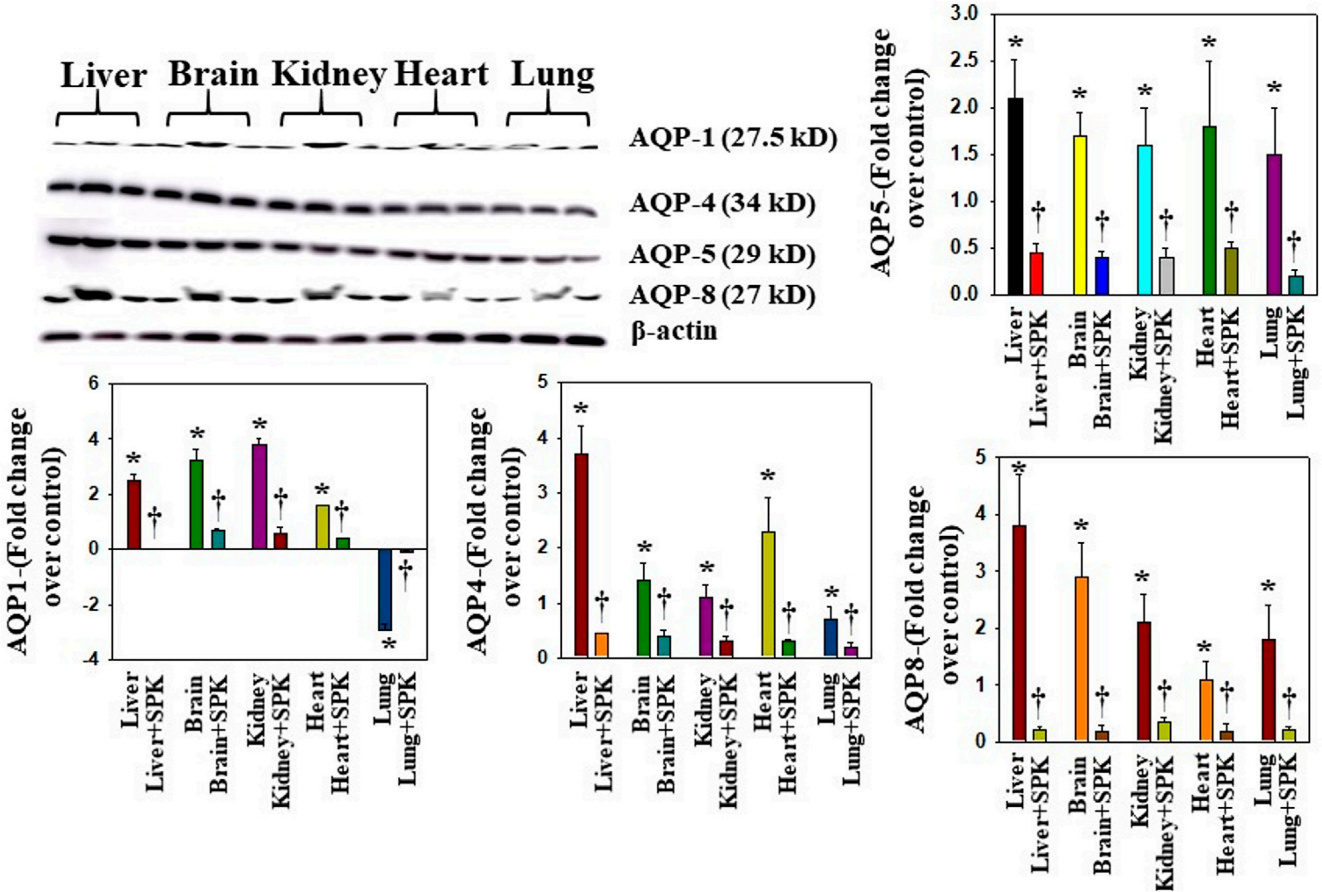
Altered AQP levels were identified in various organs post-MHV-1 inoculation. Representative immunoblots showed an increase in AQPs 1, 4, 5, and 8 in various organs, while AQP1 levels were decreased in lungs post-MHV-1. Further, treatment of MHV-1 inoculated mice with SPK (5 mg/kg) reversed AQP levels. AQP levels are normalized against β-actin. ANOVA, *n* = 4. *p < 0.05 versus control; †*p* < 0.05 verses MHV-1 infected mice. Error bars represent mean ± SEM.

Overexpression of AQPs or increased levels in plasma membrane have been shown to contribute to the development of edema or balance the intra- and extracellular water levels (Johansson et al., 1998; Ma et al., 1997; Rama Rao et al., 2010; Wittekindt and Dietl, 2019). We found that AQPs 1, 4, 5, and 8 were increased in all organs examined, while AQPs 1 was differentially regulated (Figure 6). Further, treatment with SPIKENET (5 mg/kg) reduced the levels of AQPs 4, 5, and 8 and reversed the decreased AQP1 level to normal (Figure 6). To complement the immunofluorescence observation, we also performed semiquantitaive Western blots and found simialr results with all AQPs (Figure 7), while the levels of AQPs measured by Westrn blots are slighly deferent from the immunofluorescence images. This may be due to sensitivity of the methods (i.e., the fluorescence method may be more sensitive than the Western blot). Overall, our findings strongly suggest that the altering of AQPs, likely mediated by increased oxidative stress in multiple organs, may be a crucial event in the pathogenesis of SARS-CoV-2 infection and suggests the probable role of generalized edema.

**FIGURE 8:**
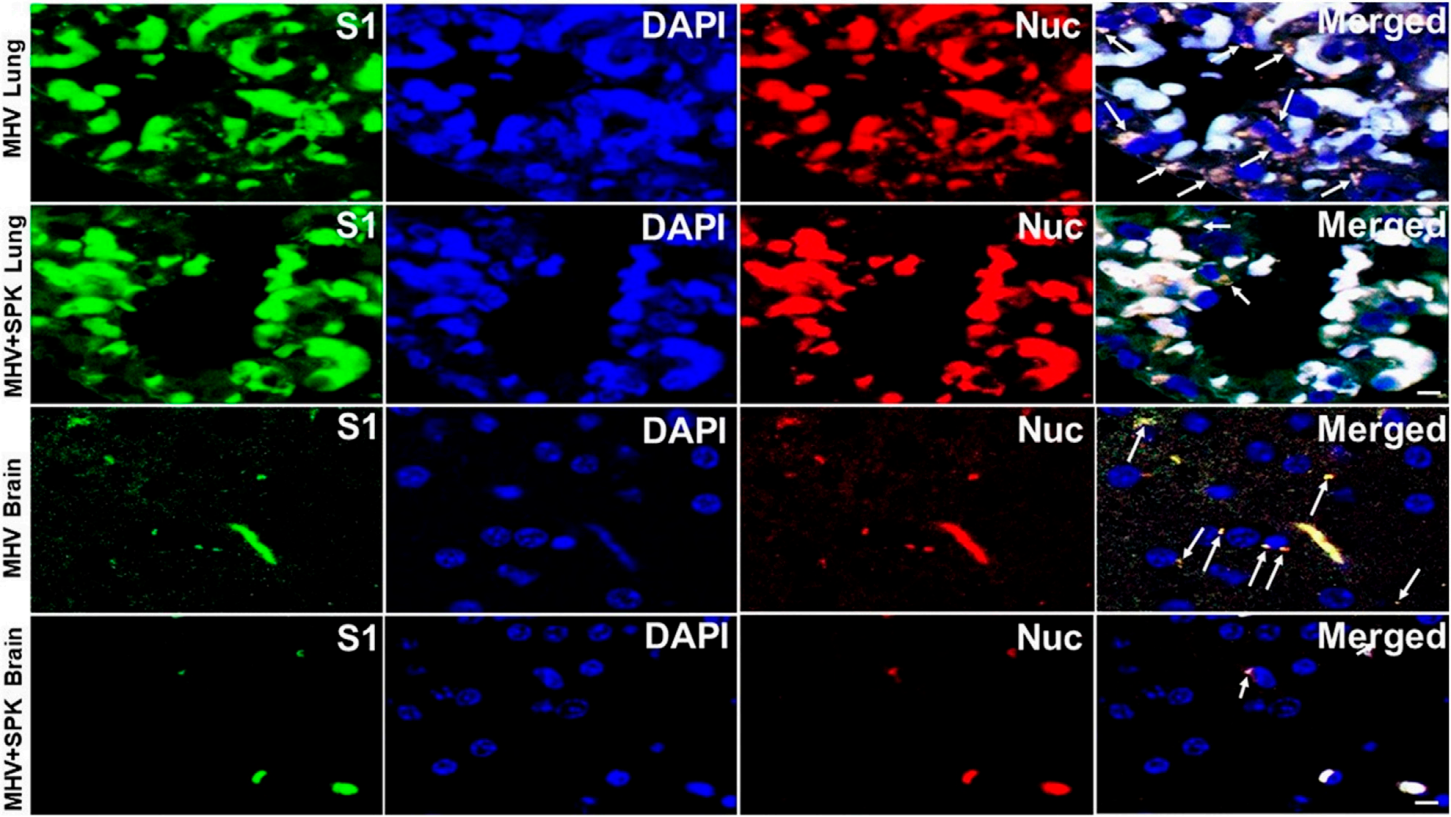
The presence of MHV-1 virus with and without SPK treatment in lung and brain. The long arrows indicate the presence of viral particles (S1 and Nucleocapsid) in the lungs and brains of MHV-1 infected mice. The short arrows indicate the reduced level of viral particles in SPK treated group *n* = 4. Scale bar = 30 μm.

**FIGURE 9:**
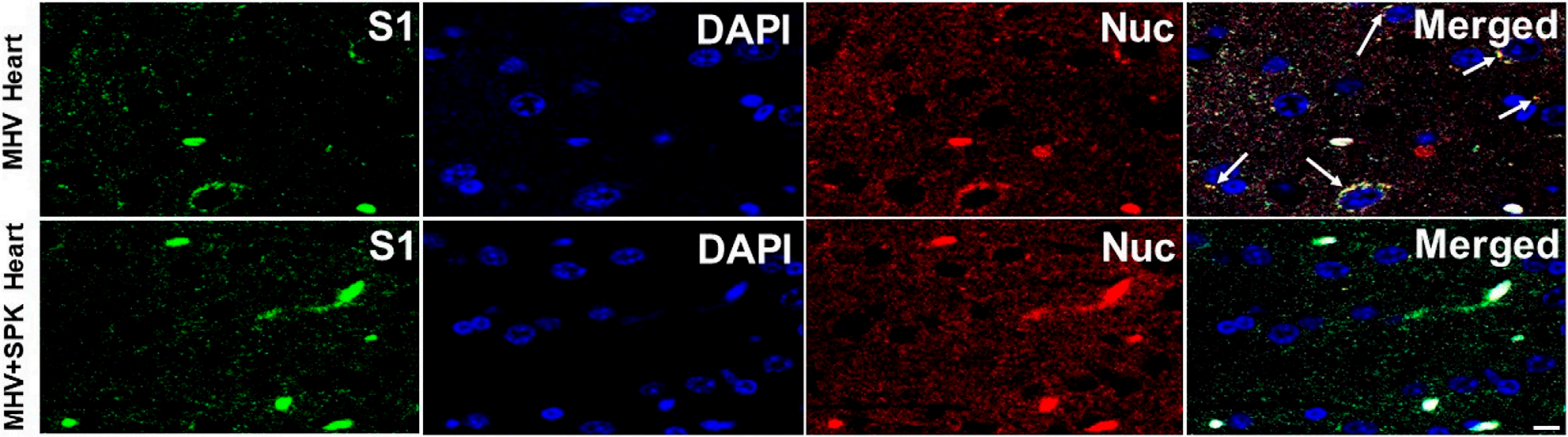
The presence of MHV-1 virus with and without SPK treatment in heart. The long arrows indicate the presence of viral particles (S1 and Nucleocapsid) in the hearts of MHV-1 infected mice. The viral particles are absent in the SPK treated group. *n* = 4. Scale bar = 30 μm.

**FIGURE 10:**
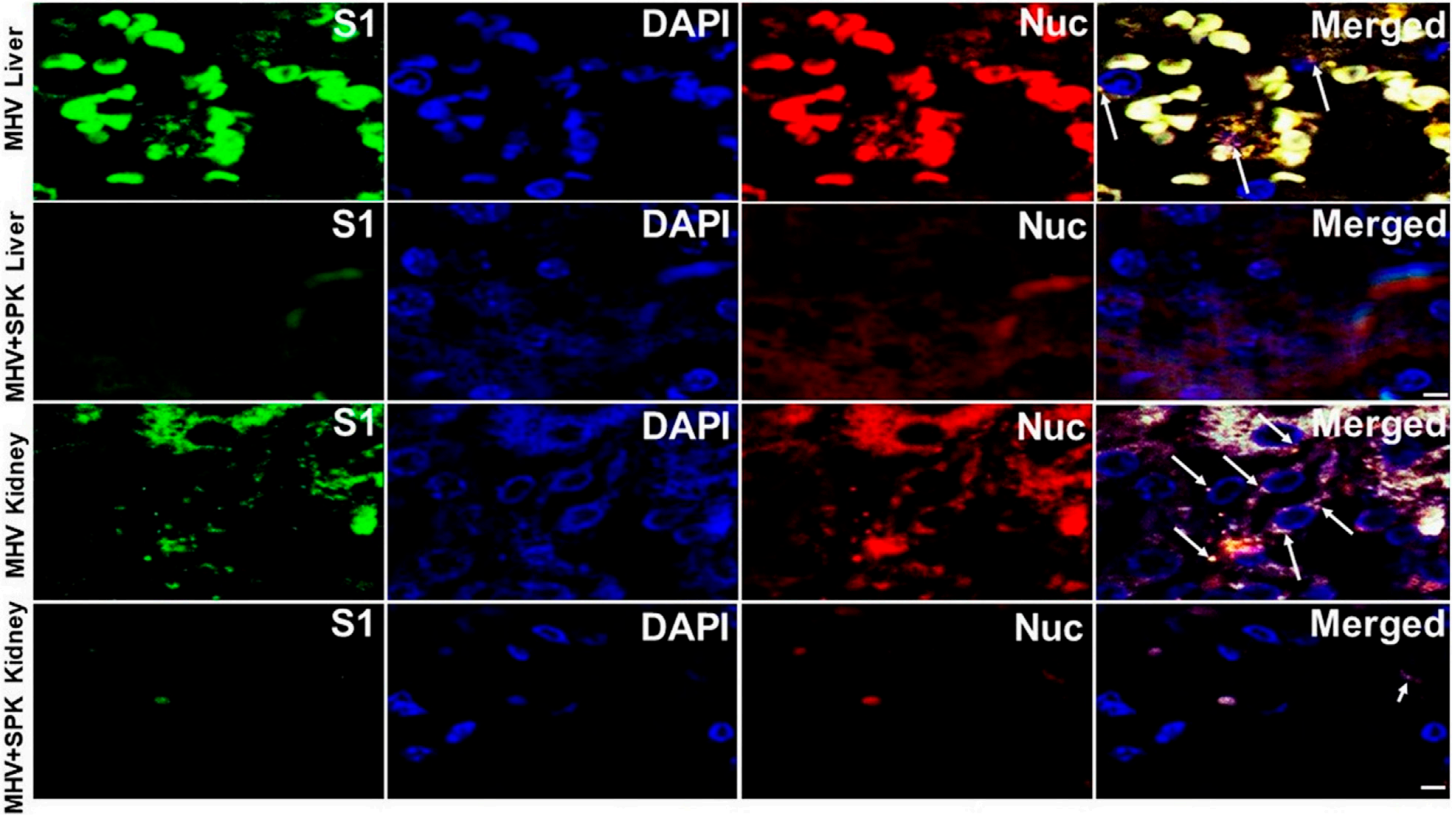
The presence of MHV-1 virus with and without SPK treatment in liver and kidney. The long arrows indicate the presence of viral particles (S1 and Nucleocapsid) in the livers and kidneys of MHV-1 infected mice. The short arrows in the kidney indicate the reduced level of viral particles in the SPK treated group, while viral particles are absent in the liver post-SPK treatment. *n* = 4. Scale bar = 30 μm.

To examine whether the SPK effect is indeed due to prevention or amilioration of viral load, we precisely exmined the presence of viral particles in all tissues with and without SPK treatment. We found that the viral particles (S1 and Nucleocapsid) are present in all organs (predominantly around the nucleus, and in the cytoplasm) in MHV-1 infeced animals. Decreased number of viral particles were identified in lung and brain, and were absent in liver, kidney and heart of MHV-1 infected mice that were treated with SPK (Figures 8–10). These findings strongly suggest that SPK prsemiquantitaive Western blots and found simialr results with allevented the viral replication although other effects (i.e., inhibition of inflammation or other factors in addition to reducing the viral load) cannot be ruled-out.

### Oxidative Stress

**FIGURE 11:**
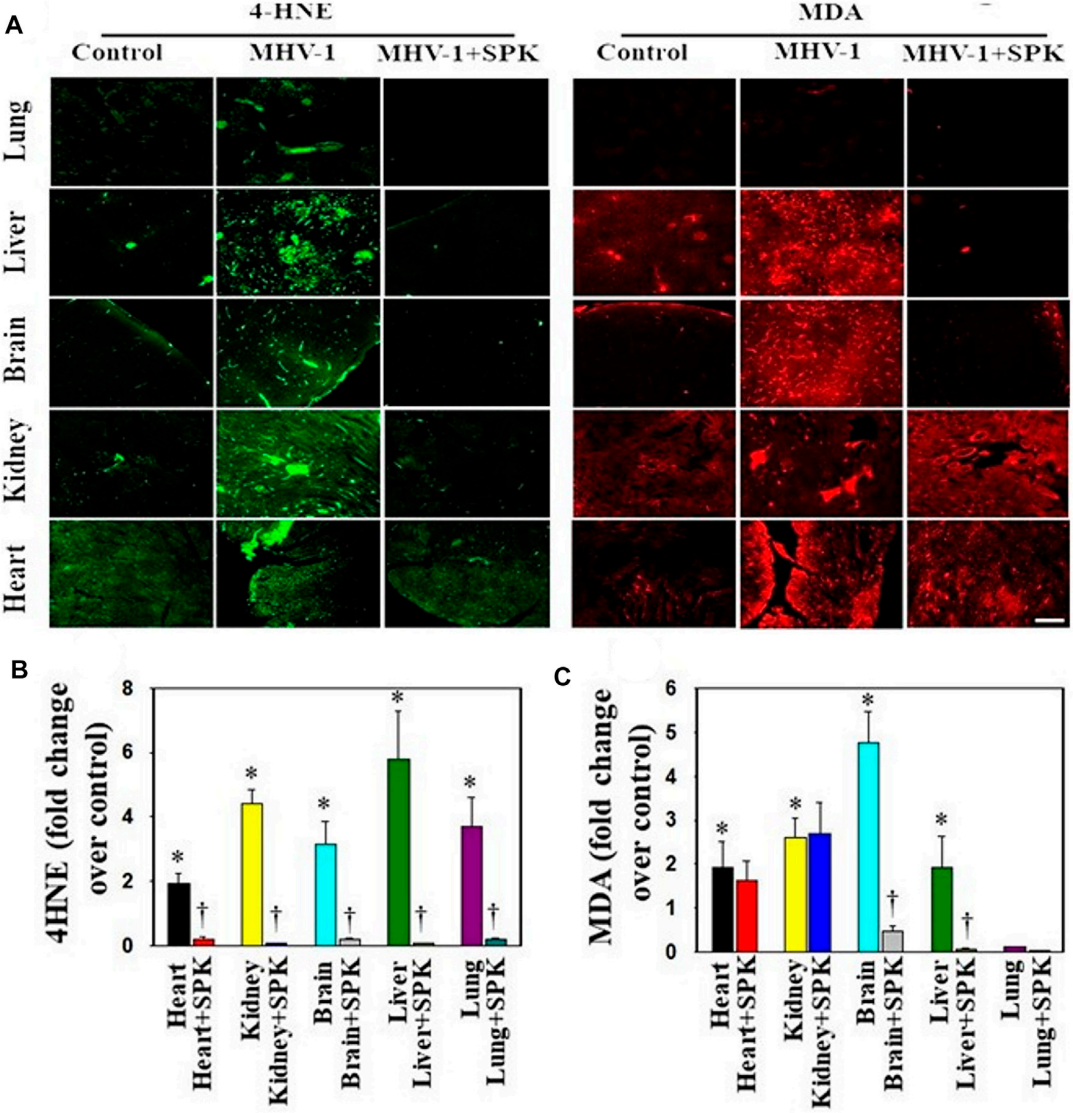
Oxidative stress post-MHV-1 infection in mice. **(A)** Representative immunofluorescence images from four individual animals show an increase in levels of 4-hydroxynonenol (4-HNE) and malondialdehyde (MDA) in lung, liver, kidney, brain and heart. Treatment of MHV-1 inoculated mice with SPK (5 mg/kg) prevented such an increase. **(B,C)** Quantitation of 4-HNE and MDA levels with and without SPK post-MHV-1 infection. ANOVA, *n* = 4. *p < 0.05 versus control; †*p* < 0.05 verses MHV-1 infected mice. Scale bar = 25 μm. Error bars represent mean ± SEM.

While alterations in the redox system have been proposed in the pathophysiology of general infection (Forcados et al., 2021), little is currently known regarding the involvement of oxidative stress in SARS-CoV-2 infection and its impact on pathophysiologic alterations. We identified lipid peroxidation-derived aldehydes, 4-hydroxynonenol (4-HNE) and malondialdehyde (MDA), in various organs of MHV-1 inoculated mice (6 days post-MHV-1) (Figure 11). Further, treatment of MHV-1 infected mice with SPIKENET (5 mg/kg) diminished such effect (Figure 11). These findings collectively suggest that OS may be a crucial factor involved in SARS-CoV-2 infection.

### Microglia and Astrocytes

**FIGURE 12:**
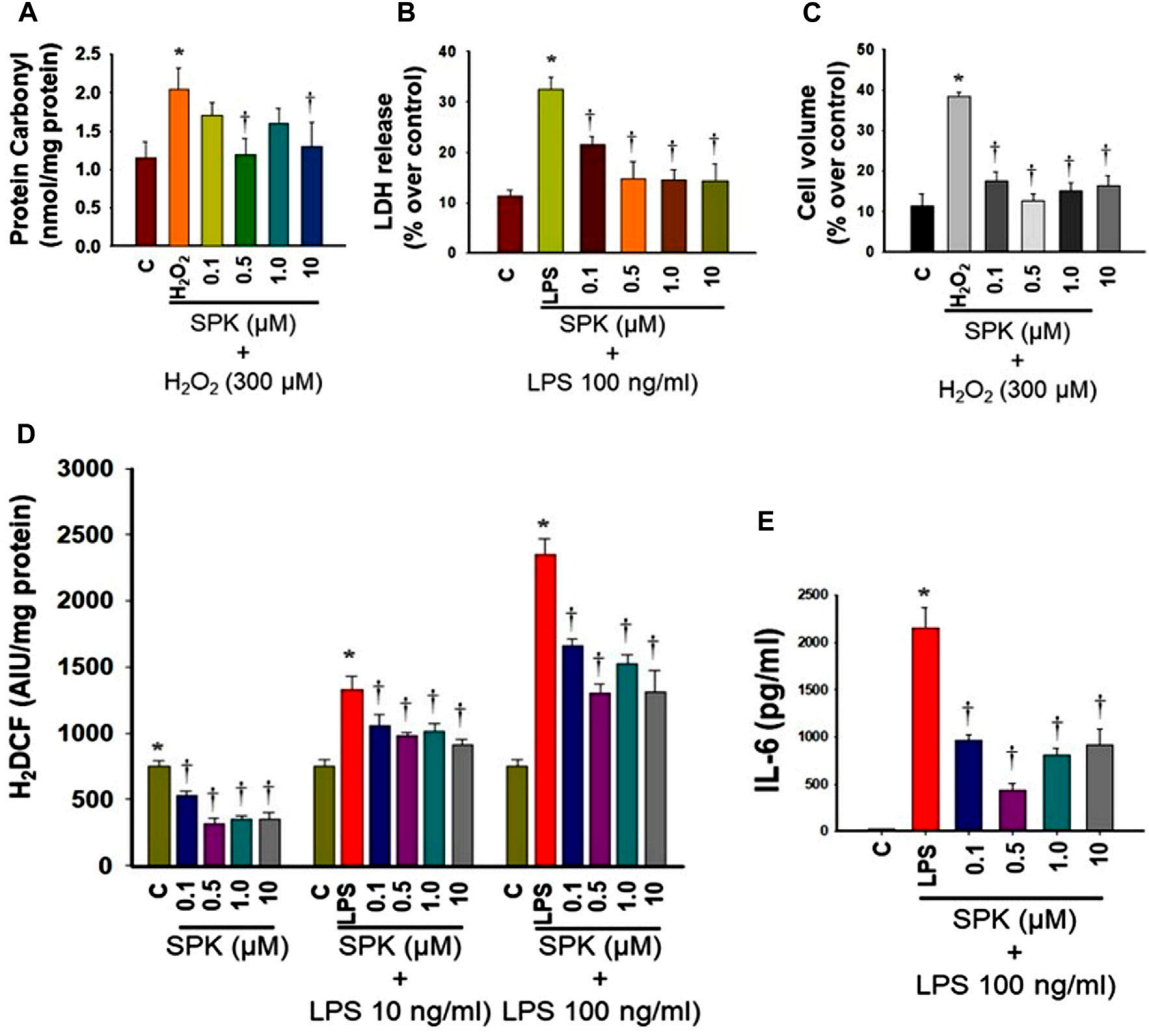
Effect of SPIKENET (SPK) on hydrogen peroxide (H_2_O_2_)-induced oxidative stress (protein carbonyl formation) (12 h) **(A)**, and LPS-induced LDH release (36 h) **(B)** in primary cultures of rat brain microglia, as well as H_2_O_2_-induced increase in cell volume (24 h) in primary cultures of rat brain astrocytes **(C)**. SPK significantly diminished these effects in glial cells (30 min post-treatment). Exposure of primary microglia to LPS (24 h) showed an increase in DCF fluorescence **(D)**, as well as an increase in IL-6 level **(E)** in cell culture medium, and such increase was diminished and blocked by treatment of cells (30 min post-treatment) with SPIKENET. C, control; LPS, lipopolysaccharide. *p < 0.05 vs. control. †*p* < 0.05 vs. LPS. C, control; AIU, arbitrary intensity units; LPS, lipopolysaccharide. Error bars represent mean ± SEM.

A hallmark of SARS-CoV-2 infection is systemic proinflammation, which is usually associated with the production of reactive oxygen species followed by oxidative stress, contributing to the development of disease progression. We, therefore, examined SPIKENET’s effects on oxidative stress and inflammation and found that SPIKENET diminished lipopolysaccharide (LPS)-induced inflammatory response, as well as oxidative stress in primary cultures of rat brain microglia (Figure 12). We also found inhibition of LPS-induced cell death (LDH release) and cell stress by SPIKENET in primary cultures of rat brain microglia, as well as inhibition of a chemically induced cell swelling in astrocyte cultures (a major event appearing in lungs post-SARS-CoV-2 infection) (Figure 12). In addition to the anti-inflammatory and anti-oxidative effects of SPIKENET, we also found that exposure of brain microglia, astrocytes and neurons to high concentrations of SPIKENET (50 and 100 μm) did not affect cell survival or mitochondrial function as measured by MTT (3-(4,5-dimethylthiazol-2-yl)-2,5-diphenyltetrazoliumbromide) assay (data not shown). Collectively these findings suggest the potential usefulness of SPIKENET to prevent SARS-CoV- 2 infection.

## Discussion

Our findings show an alteration in AQP 1, 4, 5, and 8 protein levels and associated oxidative stress, along with various degrees of tissue edema in multiple organs, which correlate well with animal survival post-MHV-1 infection. Further, SPIKENET, our newly created drug (a 15 amino acid synthetic peptide) that was designed to prevent binding of spike proteins with their receptor(s), ameliorated animal death, pathological changes, the altered protein levels of AQPs, and the formation of edema post-MHV-1. Additionally, SPIKENET also ameliorated animal death in a humanized animal model of SARS-CoV-2 infection (unpublished observation). Collectively, these findings suggest the possible involvement of aquaporins and the subsequent development of edema in various organs in the pathogenesis of COVID-19. AQP activity is highly regulated by gating or by trafficking in the plasma membrane. Phosphorylation of serine residues has been shown to modulate AQP gating (Johansson et al., 1998; Pires-Neto et al., 2016). AQPs (AQP0-AQP12) are divided into three sub-classes based on the selectiveness of the pores: orthodox aquaporins (AQPs 0, 1, 2, 4, 5, 6, and 8), aquaglyceroporins (AQPs 3, 7, 9, and 10) and unorthodox aquaporins (AQPs 11 and 12) (Agre, 2006). AQPs serve different functional purposes and are implicated in a wide variety of diseases including cancer, renal dysfunction, neurological disorders, metabolic syndrome, and cardiac diseases (Azad et al., 2021).

Lung edema can appear suddenly or may develop over a period depending on the cause (e.g., defective heart conditions, pneumonia, or exposure to toxins, viral infection, blood poisoning and sepsis, or due to certain drugs) (Towne et al., 2000; Wittekindt and Dietl, 2019), resulting in chest pain, difficulty in breathing, etc. Studies on lung diseases have shown that AQPs are involved in diseases where there is disturbance of airway surface liquid volume homeostasis, such as in asthma, chronic obstructive pulmonary disease (COPD), and acute lung injury (Towne et al., 2000; Wittekindt and Dietl, 2019). The precise mechanism involved in the development of lung edema induced by SARS-CoV-2 infection remains unknown.

As noted above, one mechanism which is actively involved in transporting water across plasma membranes is the expression of AQPs. AQPs 1, 3, 4, and 5 are predominantly expressed in the lung, particularly in the plasma membranes of endothelial, epithelial, basal, glandular, and acinar cells of the nasal epithelium, trachea, bronchi, and bronchioles (Towne et al., 2000; Wittekindt and Dietl, 2019 and references therein).

An increase in AQP1 and 5 has been observed in patients with acute lung injury and diffuse alveolar damage (Pires-Neto et al., 2016). While AQP1 levels are increased generally in most organs, its level decreased in the lung during other infections (Towne et al., 2000). Indeed, a recent study showed vascular inflammation and loss of AQP1 expression in endothelial cells, as well as increased fluid extravasation in SARS-CoV-2-infected golden Syrian Hamsters (Allnoch et al., 2021). Similarly, we identified decreased levels of AQP1 in MHV-1 inoculated mice. Further, treatment of MHV-1 infected mice with our newly created peptide, SPIKENET, reversed such a reduction, as well as diminished the level of elevated edema and animal death.

While the reason for the decline in levels of AQP-1 in our study, or the study performed in Syrian hamsters by Allnoch et al. (2021) is unclear, Allnoch et al. speculated that inflammation may be involved since there is an infiltration of macrophages in the alveolus that is known to affect intercellular junctions and might be causing the observed loss of AQP1. In general, a decrease in AQP-1 positively correlated with an increase in macrophage-derived mediators such as the generation of free radicals and cytokines, along with increased edema (Allnoch et al., 2021 and references therein). Additionally, loss of AQP1 level was also observed during hypoxic conditions (Towne et al., 2000; Wittekindt and Dietl, 2019), suggesting that hypoxia may also be a major event in the lung post-infection.

While other studies have reported decreased levels of AQP5 and associated elevation in lung edema in other viral infections (Towne et al., 2000; Zhang et al., 2018), we found elevated levels of AQPs 4, 5, and 8 in MHV-1 inoculated mice. Further, treatment with SPIKENET inhibited the increase of these AQP levels, in addition to reducing lung edema and animal death. The reason for such a contradiction between these studies and ours is unclear, but it is possible that the signaling system that is activated by SARS-CoV-2 may be different than that in other viruses studied. Collectively, these findings suggest the potential involvement of AQPs 1, 4, 5, and 8 in the development of lung edema and the subsequent progression of infection. Accordingly, our identification of altered levels of these AQPs and its contribution to the development of lung edema is crucial to better understanding the pathophysiology of SARS-Cov-2 infection.

Edema is one of the serious clinical features of nephritic and nephrotic syndrome and may cause localized puffiness to massive, generalized edema (Palmer and Alpern, 1997; Kurtzman, 2001). Abnormal accumulation of interstitial fluid in these conditions results from anomalous renal sodium retention and increased capillary wall permeability. Additionally, altered AQP expression has been strongly implicated in the pathogenesis of these syndromes (Wang et al., 2015; Valtueña et al., 2020). There are 7 AQPs in the kidney (AQP1, AQP2, AQP3, AQP4, AQP6, AQP7 and AQP11). We indeed identified increased levels of AQPs 1, 4, 5, and 8 in MHV-1 infected mice in various organs, although evidence for severe edema in the kidney in other infectious diseases is scant. Since nephritic and nephrotic syndromes are well known to induce massive, generalized edema (Kwon et al., 2009; Wang et al., 2015; Valtueña et al., 2020), it is possible that the increased level of AQPs 1, 4, 5 and 8 in MHV-1 infected mice kidneys may cause whole-body edema or it may contribute to the potentiation of infection-induced edema in various organs in COVID-19.

Edema in the liver and heart has also been implicated in disease progression in various conditions including in infection-induced injury to these organs and altered AQPs have been strongly implicated in their pathogenesis (Marinelli and LaRusso, 1997; Schrier et al., 2001; Frustaci et al., 2021). Our study also demonstrates significant edema in these organs, and treatment of these mice with SPIKENET reduced such edema. Thus, it is possible that an increase in AQPs 1, 4, 5, and 8, and the subsequent increase in edema in the liver and heart of SARS-CoV-2 infection, may contribute to the progression of COVID-19.

AQP4 has been predominantly reported to be involved in brain dysfunction due to its exclusive localization in astrocytes. Studies have also shown the potential involvement of AQPs 1, 5, and 9 in the development of brain edema in a variety of neurological conditions (Badaut et al., 2002; Papadopoulos et al., 2002). Increased AQPs, the associated edema, and the subsequent increased intracranial pressure and herniation has been strongly implicated in the development of coma and subsequent death (Agre, et al., 1997; Badaut et al., 2002). We indeed identified elevated levels of AQPs 1, 4, 5, and 8 in the brains of MHV-1 inoculated mice, along with an increase in edema, and treatment of these mice with SPIKENET exhibited a reduction in these AQPs, as well as in animal death. These findings strongly suggest the potential involvement of infection-indued brain AQPs and their involvement in edema formation and subsequent animal death in SARS-CoV-2 infection. The role of AQPs in the mechanisms of solute transport has been well established over a decade. Based on our current findings, we predict that generalized edema development may be a major event in SARS-CoV-2 infection, since alteration of water transport-related AQPs was specifically identified in this study (i.e., AQPs 1, 4, 5, 8). However, we cannot rule out the possibility of altered AQPs that regulate other solutes that may contribute to organ dysfunction. For example, AQP1 has also been proposed to transport CO_2_, glycerol, and cations under some conditions (Agre, et al., 1997; Yang .et al., 2000), but these results have been questioned (Agre, et al., 1997; Yang .et al., 2000). These aspects, in addition to its interaction with other ion channels/transporters/exchangers, need to be explored.

While we found changes in various AQPs in a particular organ, it is unclear whether all of these AQPs are involved in water transport, or they also transport other solutes/ions which may contribute to the multiorgan dysfunction observed in the current study. Further, it is not clear whether any interaction occurs in these AQPs that can regulate solute/ion movement. These aspects will be explored in future studies.

Overall our study demonstrates that AQPs 1, 4, 5, and 8 are increased in all organs examined except that AQP1 levels in the lung are reduced. Our findings on AQP1 support earlier findings showing that AQP1 levels decreased in lung endothelial cells in a humanized animal model of SARS-CoV-2 (Allnoch et al., 2021), and strongly suggest the usefulness of our model to study COVID-19. While there were limited to no expression or protein levels of one or more of the AQPs identified in various organs in normal mice, there was a significant increase in the levels of these AQPs in MHV-1 infected mice, suggesting that these AQPs may be conserved and expressed under favorable conditions. While it is not clear whether the altered levels of AQPs play a role in the pathogenesis of SARS-CoV-2 infection, our findings which demonstrate that SPIKENET prevented both animal death, as well as reversed the changes in AQP levels and edema, strongly suggest the potential role of AQPs in the mechanisms of animal death in SARS-CoV-2 infection. Therefore, targetting AQPs may be a useful approach to treat COVID-19 and diminish some of the multiorgan consequences.

## Data Availability

The raw data supporting the conclusion of this article will be made available by the authors, without undue reservation.

## Abbreviations

4-HNE, 4-hydroxynonenol; ACE2, angiotensin-converting enzyme 2; AST, aspartate aminotransaminase; ALT, alanine aminotransaminase; ALP, alkaline phosphatase; AQPs, aquaporins; CEACAM1 or CCM, carcinoembryonic antigen-related cell adhesion molecule 1; COPD, chronic obstructive pulmonary disease; COVID-19, coronavirus disease-19; DMEM, dulbecco’s modified eagle medium; GFAP, glial fibrillary acidic protein; LPS, lipopolysaccharide; MAS, multiple sequence alignments; MD, molecular dynamics; MDA, malondialdehyde; MHV- 1, murine hepatitis virus-1; NTD, N-terminal domain; RBD, receptor-binding domain; RMSD, root mean square deviation; S1, spike glycoprotein-1; SARS-CoV-2, severe acute respiratory syndrome coronavirus 2; SPK, SPIKENET; VMD, visual molecular dynamics.

## References

[B1] Ackermann M. Verleden S. E. Kuehnel M. Haverich A. Welte T. Laenger F. (2020). Pulmonary Vascular Endothelialitis, Thrombosis, and Angiogenesis in Covid-19. N. Engl. J. Med. 383 (2), 120–128. 10.1056/NEJMoa2015432 32437596 PMC7412750

[B2] Agostini M. L. Andres E. L. Sims A. C. Graham R. L. Sheahan T. P. Lu X. (2018). Coronavirus Susceptibility to the Antiviral Remdesivir (GS-5734) Is Mediated by the Viral Polymerase and the Proofreading Exoribonuclease. mBio 9 (2), e00221–18. 10.1128/mBio.00221-18 29511076 PMC5844999

[B3] Agre P. Lee M. D. Devidas S. Guggino W. B. (1997). Aquaporins and Ion Conductance. Science 275, 5305. 9045617

[B4] Agre P. (2006). “The Aquaporin Water Channels,” in , 3, 5–13.Proc. Am. Thorac. Soc. 1 16493146 10.1513/pats.200510-109JH PMC2658677

[B5] Allnoch L. Beythien G. Leitzen E. Becker K. Kau p. F. J. Stanelle-Bertram S. (2021). Vascular Inflammation Is Associated with Loss of Aquaporin 1 Expression on Endothelial Cells and Increased Fluid Leakage in SARS-CoV-2 Infected Golden Syrian Hamsters. Viruses 13 (4), 639. 33918079 10.3390/v13040639 PMC8069375

[B6] Azad A. K. Raihan T. Ahmed J. Hakim A. Emon T. H. Chowdhury P. A. (2021). Human Aquaporins: Functional Diversity and Potential Roles in Infectious and Non-infectious Diseases. Front. Genet. 12, 654865. 10.3389/fgene.2021.654865 33796134 PMC8007926

[B7] Badaut J. Lasbennes F. Magistretti P. J. Regli L. (2002). Aquaporins in Brain: Distribution, Physiology, and Pathophysiology. J. Cereb. Blood. Flow. Metab. 22 (4), 367–378. 10.1097/00004647-200204000-00001 11919508

[B8] Caldera-Crespo L. A. Paidas M. J. Roy S. Schulman C. I. Kenyon N. S. Daunert S. (2021). Experimental Models of COVID-19. Frontiers Cellular and Infection Microbiology. Clin. Microbiol. Sect. 11, 792584. 10.3389/fcimb PMC8791197 35096645

[B9] Chen N. Zhou M. Dong X. Qu J. Gong F. Han Y. (2020). Epidemiological and Clinical Characteristics of 99 Cases of 2019 Novel Coronavirus Pneumonia in Wuhan, China: a Descriptive Study. Lancet 395 (10223), 507–513. 32007143 10.1016/S0140-6736(20)30211-7 PMC7135076

[B10] Creed M. A. Ballesteros E. L. J. G. Jr Jr L. J. G. Imitola J. (2020). Mild COVID-19 Infection Despite Chronic B Cell Depletion in a Patient with Aquaporin-4-Positive Neuromyelitis Optica Spectrum Disorder. Mult. Scler. Relat. Disord. 44, 102199. 10.1016/j.msard.2020.102199 32554285 PMC7236713

[B11] De Albuquerque N. Baig E. Ma X. Zhang J. He W. Rowe A. (2006). Murine Hepatitis Virus Strain 1 Produces a Clinically Relevant Model of Severe Acute Respiratory Syndrome in A/J Mice. J. Virol. 80 (21), 10382–10394. 10.1128/jvi.00747-06 17041219 PMC1641767

[B12] Duan L. Zheng Q. Zhang H. Niu Y. Lou Y. Wang H. (2020). The SARS-CoV-2 Spike Glycoprotein Biosynthesis, Structure, Function, and Antigenicity: Implications for the Design of Spike-Based Vaccine Immunogens. Front. Immunol. 11, 576622. 10.3389/fimmu.2020.576622 33117378 PMC7575906

[B13] Ducis I. Norenberg L. O. Norenberg M. D. (1990). The Benzodiazepine Receptor in Cultured Astrocytes from Genetically Epilepsy-Prone Rats. Brain Res. 531, 318–321. 10.1016/0006-8993(90)90793-b 1963103

[B14] El Zowalaty M. E. Järhult J. D. (2020). From SARS to COVID-19: A Previously Unknown SARS- Related Coronavirus (SARS-CoV-2) of Pandemic Potential Infecting Humans - Call for a One Health Approach. One Health 9, 100124. 10.1016/j.onehlt.2020.100124 32195311 PMC7075990

[B15] Flanary B. E. Streit W. J. (2006). Alpha-tocopherol (Vitamin E) Induces Rapid, Nonsustained Proliferation in Cultured Rat Microglia. Glia 53, 669–674. 10.1002/glia.20313 16419088

[B16] Forcados G. E. Muhammad A. Oladipo O. O. Makama S. Meseko C. A. (2021). Metabolic Implications of Oxidative Stress and Inflammatory Process in SARS-CoV-2 Pathogenesis: Therapeutic Potential of Natural Antioxidants. Front. Cell. Infect. Microbiol. 11, 654813. 10.3389/fcimb.2021.654813 34123871 PMC8188981

[B17] Fragoso D. C. Marx C. Dutra B. G. da Silva C. J. da Silva P. M. Martins Maia Junior A. C. (2021). Covid-19 as a Cause of Acute Neonatal Encephalitis and Cerebral Cytotoxic Edema. Pediatr. Infect. Dis. J. 40 (7), e270–e271. 33902082 10.1097/INF.0000000000003145

[B18] Frustaci A. Letizia C. Chimenti C. Verardo R. Alfarano M. Scialla R. (2021). Myocardial Aldosterone Receptor and Aquaporin 1 Up-Regulation Is Associated with Cardiomyocyte Remodeling in Human Heart Failure. J. Clin. Med. 10 (21), 4854. 10.3390/jcm10214854 34768373 PMC8585058

[B19] Gu J. Gong E. Zhang B. Zheng J. Gao Z. Zhong Y. (2005). Multiple Organ Infection and the Pathogenesis of SARS. J. Exp. Med. 202 (3), 415–424. 10.1084/jem.20050828 16043521 PMC2213088

[B20] Henderson L. A. Canna S. W. Friedman K. G. Gorelik M. Lapidus S. K. Bassiri H. (2021). American College of Rheumatology Clinical Guidance for Multisystem Inflammatory Syndrome in Children Associated with SARS-CoV-2 and Hyperinflammation in Pediatric COVID-19: Version 2. Arthritis Rheumatol. 73 (4), e13–e29. 10.1002/art.41616 33277976 PMC8559788

[B21] Huang Y. Yang C. Xu X. F. Xu W. Liu S. W. (2020). Structural and Functional Properties of SARS-CoV-2 Spike Protein: Potential Antivirus Drug Development for COVID-19. Acta Pharmacol. Sin. 41 (9), 1141–1149. 10.1038/s41401-020-0485-4 32747721 PMC7396720

[B22] Jackson C. B. Farzan M. Chen B. Choe H. (2022). Mechanisms of SARS-CoV-2 Entry into Cells. Nat. Rev. Mol. Cell. Biol. 23 (1), 3–20. 10.1038/s41580-021-00418-x 34611326 PMC8491763

[B23] Jayakumar A. R. Tong X. Y. Curtis K. M. Ruiz-Cordero R. Abreu M. T. Norenberg M. D. (2014a). Increased Toll-like Receptor 4 in Cerebral Endothelial Cells Contributes to the Astrocyte Swelling and Brain Edema in Acute Hepatic Encephalopathy. J. Neurochem. 128 (6), 890–903. 10.1111/jnc.12516 24261962 PMC3951576

[B24] Jayakumar A. R. Tong X. Y. Shamaladevi N. Barcelona S. Gaidosh G. Agarwal A. (2017). Defective Synthesis and Release of Astrocytic Thrombospondin-1 Mediates the Neuronal TDP-43 Proteinopathy, Resulting in Defects in Neuronal Integrity Associated with Chronic Traumatic Encephalopathy: *In Vitro* Studies. J. Neurochem. 140 (4), 645–661. 10.1111/jnc.13867 27735996

[B25] Jayakumar A. R. Valdes V. Tong X. Y. Shamaladevi N. Gonzalez W. Norenberg M. D. (2014b). Sulfonylurea Receptor 1 Contributes to the Astrocyte Swelling and Brain Edema in Acute Liver Failure. Transl. Stroke. Res. 5 (1), 28–37. 24443056 10.1007/s12975-014-0328-z PMC4714761

[B26] Johansson I. Karlsson M. Shukla V. K. Chrispeels M. J. Larsson C. Kjellbom P. (1998). Water Transport Activity of the Plasma Membrane Aquaporin PM28A Is Regulated by Phosphorylation. Plant Cell. 10 (3), 451–459. 10.1105/tpc.10.3.451 9501117 PMC144000

[B27] Jose R. J. Manuel A. (2020). COVID-19 Cytokine Storm: the Interplay between Inflammation and Coagulation. Lancet. Respir. Med. 8 (6), e46–ee7. 10.1016/S2213-2600(20)30216-2 32353251 PMC7185942

[B28] Juurlink B. H. Hertz L. (1985). Plasticity of Astrocytes in Primary Cultures: an Experimental Tool and a Reason for Methodological Caution. Dev. Neurosci. 7, 263–277. 10.1159/000112295 3915290

[B29] Khanolkar A. Hartwig S. M. Haag B. A. Meyerholz D. K. Epping L. L. Haring J. S. (2009). Protective and Pathologic Roles of the Immune Response to Mouse Hepatitis Virus Type 1: Implications for Severe Acute Respiratory Syndrome. J. Virol. 83 (18), 9258–9272. 10.1128/jvi.00355-09 19570864 PMC2738266

[B30] Kurtzman N. A. (2001). Nephritic Edema. Semin. Nephrol. 21 (3), 257–261. 10.1053/snep.2001.21653 11320490

[B31] Kwon T. H. Nielsen J. Møller H. B. Fenton R. A. Nielsen S. Frøkiaer J. (2009). Aquaporins in the Kidney. Handb. Exp. Pharmacol. 190, 95–132. 10.1007/978-3-540-79885-9_5 19096774

[B32] Larsen C. P. Bourne T. D. Wilson J. D. Saqqa O. Sharshir M. A. (2020). Collapsing Glomerulopathy in a Patient with COVID-19. Kidney. Int. Rep. 5 (6), 935–939. 10.1016/j.ekir.2020.04.002 32292867 PMC7142700

[B33] Leibowitz J. L. Srinivasa R. Williamson S. T. Chua M. M. Liu M. Wu S. (2010). Genetic Determinants of Mouse Hepatitis Virus Strain 1 Pneumovirulence. J. Virol. 84 (18), 9278–9291. 10.1128/JVI.00330-10 20631137 PMC2937641

[B34] Lopes-Pacheco M. Silva P. L. Cruz F. F. Battaglini D. Robba C. Pelosi P. (2021). Pathogenesis of Multiple Organ Injury in COVID-19 and Potential Therapeutic Strategies. Front. Physiol. 12, 593223. 10.3389/fphys.2021.593223 33584343 PMC7876335

[B35] Ma T. Yang B. Verkman A. S. (1997). Cloning of a Novel Water and Urea-Permeable Aquaporin from Mouse Expressed Strongly in Colon, Placenta, Liver, and Heart. Biochem. Biophys. Res. Commun. 240 (2), 324–328. 10.1006/bbrc.1997.7664 9388476

[B36] Marinelli R. A. LaRusso N. F. (1997). Aquaporin Water Channels in Liver: Their Significance in Bile Formation. Hepatology 26 (5), 1081–1084. 10.1002/hep.510260539 9362345

[B37] Matsuishi Y. Mathis B. J. Shimojo N. Subrina J. Okubo N. Inoue Y. (2021). Severe COVID-19 Infection Associated with Endothelial Dysfunction Induces Multiple Organ Dysfunction: A Review of Therapeutic Interventions. Biomedicines 9 (3), 279. 10.3390/biomedicines9030279 33801921 PMC7999560

[B38] Menter T. Haslbauer J. D. Nienhold R. Savic S. Hopfer H. Deigendesch N. (2020). Postmortem Examination of COVID-19 Patients Reveals Diffuse Alveolar Damage with Severe Capillary Congestion and Variegated Findings in Lungs and Other Organs Suggesting Vascular Dysfunction. Histopathology 77 (2), 198–209. 10.1111/his.14134 32364264 PMC7496150

[B39] Mitchell W. B. (2020). Thromboinflammation in COVID-19 Acute Lung Injury. Paediatr. Respir. Rev. 35, 20–24. 10.1016/j.prrv.2020.06.004 32653469 PMC7289106

[B40] Mokhtari T. Hassani F. Ghaffari N. Ebrahimi B. Yarahmadi A. Hassanzadeh G. (2020). COVID-19 and Multiorgan Failure: A Narrative Review on Potential Mechanisms. J. Mol. Histol. 51 (6), 613–628. 10.1007/s10735-020-09915-3 33011887 PMC7533045

[B41] Paidas M. J. Mohamed A. B. Norenberg M. D. Saad A. Barry A. F. Colon C. (2021). Multi-Organ Histopathological Changes in a Mouse Hepatitis Virus Model of COVID-19. Viruses 13 (9), 1703. 10.3390/v13091703 34578284 PMC8473123

[B42] Palmer B. F. Alpern R. J. (1997). Pathogenesis of Edema Formation in the Nephrotic Syndrome. Kidney. Int. Suppl. 59, S21–S27. 9185099

[B43] Papadopoulos M. C. Krishna S. Verkman A. S. (2002). Aquaporin Water Channels and Brain Edema. Mt. Sinai. J. Med. 69 (4), 242–248. 12357265

[B44] Peng G. Sun D. Rajashankar K. R. Qian Z. Holmes K. V. Li F. (2011). Crystal Structure of Mouse Coronavirus Receptor-Binding Domain Complexed with its Murine Receptor. Proc. Natl. Acad. Sci. U. S. A. 108 (26), 10696–10701. 10.1073/pnas.1104306108 21670291 PMC3127895

[B45] Pires-Neto R. C. Del Carlo Bernardi F. Alves de Araujo P. Mauad T. Dolhnikoff M. (2016). The Expression of Water and Ion Channels in Diffuse Alveolar Damage Is Not Dependent on DAD Etiology. PLoS One 11 (11), e0166184. 10.1371/journal.pone.0166184 27835672 PMC5106024

[B46] Puntmann V. O. Carerj M. L. Wieters I. Fahim M. Arendt C. Hoffmann J. (2020). Outcomes of Cardiovascular Magnetic Resonance Imaging in Patients Recently Recovered from Coronavirus Disease 2019 (COVID-19). JAMA. Cardiol. 5 (11), 1265–1273. 10.1001/jamacardio.2020.3557 32730619 PMC7385689

[B47] Rahimi N. (2020). C-type Lectin CD209L/L-SIGN and CD209/DC-SIGN: Cell Adhesion Molecules Turned to Pathogen Recognition Receptors. Biol. (Basel) 10 (1), 1. 10.3390/biology10010001 PMC7822156 33375175

[B48] Rama Rao K. V. Jayakumar A. R. Tong X. Curtis K. M. Norenberg M. D. (2010). Brain Aquaporin-4 in Experimental Acute Liver Failure. J. Neuropathol. Exp. Neurol. 69 (9), 869–879. 20720509 10.1097/NEN.0b013e3181ebe581 PMC4737434

[B49] Reichard R. R. Kashani K. B. Boire N. A. Constantopoulos E. Guo Y. Lucchinetti C. F. (2020). Neuropathology of COVID-19: a Spectrum of Vascular and Acute Disseminated Encephalomyelitis (ADEM)-like Pathology. Acta. Neuropathol. 140, 1–6. 32449057 10.1007/s00401-020-02166-2 PMC7245994

[B50] Robba C. Battaglini D. Pelosi P. Rocco P. R. M. (2020). Multiple Organ Dysfunction in SARS-CoV-2: MODS-CoV-2. Expert Rev. Respir. Med. 14 (9), 865–868. 10.1080/17476348.2020.1778470 32567404 PMC7441756

[B51] Rump K. Adamzik M. (2018). Function of Aquaporins in Sepsis: a Systematic Review. Cell. Biosci. 8, 10. 29449936 10.1186/s13578-018-0211-9 PMC5807818

[B52] Schrier R. W. Cadnapaphornchai M. A. Ohara M. (2001). Water Retention and Aquaporins in Heart Failure, Liver Disease and Pregnancy. J. R. Soc. Med. 94 (6), 265–269. 10.1177/014107680109400603 11387413 PMC1281519

[B53] Shanmugaraj B. Siriwattananon K. Wangkanont K. Phoolcharoen W. (2020). Perspectives on Monoclonal Antibody Therapy as Potential Therapeutic Intervention for Coronavirus Disease-19 (COVID-19). Asian Pac J. Allergy Immunol. 38 (1), 10–18. 10.12932/AP-200220-0773 32134278

[B54] Thakur V. Ratho R. K. Kumar P. Bhatia S. K. Bora I. Mohi G. K. (2021). Multi-Organ Involvement in COVID-19: Beyond Pulmonary Manifestations. J. Clin. Med. 10 (3), 446. 10.3390/jcm10030446 33498861 PMC7866189

[B55] Thépaut M. Luczkowiak J. Vivès C. Labiod N. Bally I. Lasala F. (2021). DC/L- SIGN Recognition of Spike Glycoprotein Promotes SARS-CoV-2 Trans-infection and Can Be Inhibited by a Glycomimetic Antagonist. PLoS Pathog. 17 (5), e1009576. 34015061 10.1371/journal.ppat.1009576 PMC8136665

[B56] Tian J. Middleton B. Kaufman D. L. (2021). GABAA-receptor Agonists Limit Pneumonitis and Death in Murine Coronavirus-Infected Mice. Viruses 13 (6), 966. 10.3390/v13060966 34071034 PMC8224554

[B57] Towne J. E. Harrod K. S. Krane C. M. Menon A. G. (2000). Decreased Expression of Aquaporin (AQP)1 and AQP5 in Mouse Lung after Acute Viral Infection. Am. J. Respir. Cell. Mol. Biol. 22 (1), 34–44. 10.1165/ajrcmb.22.1.3818 10615063

[B58] Trbojević-Akmačić I. Petrović T. Lauc G. (2021). SARS-CoV-2 S Glycoprotein Binding to Multiple Host Receptors Enables Cell Entry and Infection. Glycoconj J. 38, 611–623. 10.1007/s10719-021-10021-z 34542788 PMC8450557

[B59] Tyagi S. C. Singh M. (2021). Multi-organ Damage by Covid-19: Congestive (Cardio-pulmonary) Heart Failure, and Blood-Heart Barrier Leakage. Mol. Cell. Biochem. 476 (4), 1891–1895. 10.1007/s11010-021-04054-z 33483858 PMC7822399

[B60] Valtueña J. Ruiz-Sánchez D. Volo V. Manchado-López P. Garayar-Cantero M. (2020). Acral Edema during the COVID-19 Pandemic. Int. J. Dermatol. 59 (9), 1155–1157. 32608503 10.1111/ijd.15025 PMC7361594

[B61] Verkman A. S. Mitra A. K. (2000). Structure and Function of Aquaporin Water Channels. Am. J. Physiol. Ren. Physiol. 278 (1), F13–F28. 10.1152/ajprenal.2000.278.1.f13 10644652

[B62] Wang Y. Bu J. Zhang Q. Chen K. Zhang J. Bao X. (2015). Expression Pattern of Aquaporins in Patients with Primary Nephrotic Syndrome with Edema. Mol. Med. Rep. 12 (4), 5625–5632. 10.3892/mmr.2015.4209 26261083 PMC4581814

[B63] Wang Y. Liu S. Liu H. Li W. Lin F. Jiang L. (2020). SARS-CoV-2 Infection of the Liver Directly Contributes to Hepatic Impairment in Patients with COVID-19. J. Hepatol. 73 (4), 807–816. 10.1016/j.jhep.2020.05.002 32437830 PMC7211738

[B64] Wiggli B. Imhof E. Meier C. A. Laifer G. (2013). Water, Water, Everywhere. Acute Parvovirus B19 Infection. Lancet 381 (9868), 776. 10.1016/S0140-6736(12)61894-7 23472922

[B65] Wittekindt O. H. Dietl P. (2019). Aquaporins in the Lung. Pflugers Arch. 471 (4), 519–532. 30397774 10.1007/s00424-018-2232-y PMC6435619

[B66] Xu Z. Shi L. Wang Y. Zhang J. Huang L. Zhang C. (2020). Pathological Findings of COVID-19 Associated with Acute Respiratory Distress Syndrome. Pflugers Arch. 8 (4), 420–422. 10.1007/s00424-018-2232-y PMC7164771 32085846

[B67] Yang B. Fukuda N. van Hoek A. Matthay M. A. Ma T. Verkman A. S. (2000). Carbon Dioxide Permeability of Aquaporin-1 Measured in Erythrocytes and Lung of Aquaporin-1 Null Mice and in Reconstituted Proteoliposomes. J. Biol. Chem. 275 (4), 2686–2692. 10644730 10.1074/jbc.275.4.2686

[B68] Yang N. Shen H. M. (2020). Targeting the Endocytic Pathway and Autophagy Process as a Novel Therapeutic Strategy in COVID-19. Int. J. Biol. Sci. 16 (10), 1724–1731. 10.7150/ijbs.45498 32226290 PMC7098027

[B69] Zaim S. Chong J. H. Sankaranarayanan V. Harky A. (2020). COVID-19 and Multiorgan Response. Curr. Probl. Cardiol. 45 (8), 100618. 10.1016/j.cpcardiol.2020.100618 32439197 PMC7187881

[B70] Zhang J. Yan M. Gu W. Chen A. Liu J. Li L. (2018). Downregulation of Aquaporins (AQP1 and AQP5) and Na,K-ATPase in Porcine Reproductive and Respiratory Syndrome Virus-Infected Pig Lungs. Inflammation 41 (3), 1104–1114. 10.1007/s10753-018-0762-2 29532265

[B71] Zhang Q. Xiang R. Huo S. Zhou Y. Jiang S. Wang Q. (2021). Molecular Mechanism of Interaction between SARS-CoV-2 and Host Cells and Interventional Therapy. Signal Transduct. Target Ther. 6 (1), 233. 10.1038/s41392-021-00653-w 34117216 PMC8193598

[B72] Zhao W. Li H. Li J. Xu B. Xu J. (2022). The Mechanism of Multiple Organ Dysfunction Syndrome in Patients with COVID-19. J. Med. Virol. 94 (5), 1886–1892. 10.1002/jmv.27627 35088424 PMC9015222

[B73] Zhou F. Yu T. Du R. Fan G. Liu Y. Liu Z. (2020). Clinical Course and Risk Factors for Mortality of Adult Inpatients with COVID-19 in Wuhan, China: a Retrospective Cohort Study. Lancet 395 (10229), 1054–1062. 10.1016/S0140-6736(20)30566-3 32171076 PMC7270627

